# Gastrointestinal Sequelae of COVID-19: Investigating Post-Infection Complications—A Systematic Review

**DOI:** 10.3390/v16101516

**Published:** 2024-09-25

**Authors:** Ibrahim Mohammed, Sudharsan Podhala, Fariha Zamir, Shamha Shiyam, Abdel Rahman Salameh, Zoya Salahuddin, Huda Salameh, Chaehyun Kim, Zena Sinan, Jeongyeon Kim, Deema Al-Abdulla, Sa’ad Laws, Malik Mushannen, Dalia Zakaria

**Affiliations:** 1 Department of Medicine, Albany Medical College, New York, NY 12208, USA; mohammi3@amc.edu; 2 Department of Medical Education, Weill Cornell Medicine-Qatar, Doha P.O. Box 24144, Qatarzos4001@qatar-med.cornell.edu (Z.S.); zes4002@qatar-med.cornell.edu (Z.S.);; 3 School of Medicine, Royal College of Surgeons in Ireland, D02 YN77 Dublin, Ireland; abdelrahmansala22@rcsi.ie; 4 Health Sciences Library, Weill Cornell Medicine-Qatar, Doha P.O. Box 24144, Qatar; 5 Department of Medicine, New York-Presbyterian Brooklyn Methodist Hospital, New York, NY 11215, USA; 6 Department of Premedical Education, Weill Cornell Medicine-Qatar, Doha P.O. Box 24144, Qatar

**Keywords:** COVID-19, SARS-CoV-2, post-COVID sequelae, long-COVID, digestive tract gastrointestinal tract, COVID-19 complications, COVID-19 and gut

## Abstract

Gastrointestinal (GI) complications are significant manifestations of COVID-19 and are increasingly being recognized. These complications range from severe acute pancreatitis to colitis, adding complexity to diagnosis and management. A comprehensive database search was conducted using several databases. Our inclusion criteria encompassed studies reporting severe and long-term GI complications of COVID-19. Digestive disorders were categorized into infections, inflammatory conditions, vascular disorders, structural abnormalities, other diagnoses, and undiagnosed conditions. Of the 73 studies that were selected for full-text review, only 24 met our inclusion criteria. The study highlights a broad range of gastrointestinal complications following COVID-19 infection (excluding liver complications, which are examined separately), including inflammatory conditions, such as ulcerative colitis (UC), acute pancreatitis, and multisystem inflammatory syndrome in children (MIS-C). Other GI complications were reported such as vascular disorders, including diverse thrombotic events and structural abnormalities, which ranged from bowel perforations to adhesions. Additionally, undiagnosed conditions like nausea and abdominal pain were prevalent across different studies involving 561 patients. The findings emphasize the substantial impact of COVID-19 on the GI tract. Ongoing research and monitoring are crucial to understanding the long-term effects and developing effective management strategies for these complications.

## 1. Introduction

Gastrointestinal complications have emerged as significant manifestations of COVID-19 infection, with symptoms such as abdominal pain, nausea, vomiting, diarrhea, and constipation garnering attention in clinical settings [1]. The involvement of the gastrointestinal tract in COVID-19 has been increasingly recognized, shedding light on the diverse array of symptoms that can accompany the viral infection [2]. Studies have reported cases of severe acute pancreatitis, gastric perforation, and colitis associated with COVID-19 infection, highlighting the broad spectrum of complications that can arise [3,4,5]. These complications highlight the importance of considering COVID-19 in the differential diagnosis of patients presenting with abdominal symptoms, as the viral infection can be present in the context of several gastrointestinal pathologies, leading to diagnostic challenges and potential delays in appropriate management [6].

Furthermore, the interplay between COVID-19 and gastrointestinal manifestations extends beyond mere symptomatology, with reports of bowel ischemia, intestinal perforation, and thromboses following COVID-19 infection or vaccination [7,8,9]. Such complications emphasize the systemic nature of the disease and the need for a comprehensive understanding of its impact on various organ systems, including the gastrointestinal tract. Additionally, the presence of acute abdominal pain in COVID-19 patients, sometimes even in the absence of a fever, highlights the atypical presentations that healthcare providers need to be aware of [10,11]. These atypical presentations not only pose diagnostic challenges but also stress the importance of a high index of suspicion for COVID-19 in patients presenting with gastrointestinal symptoms, even in the absence of typical respiratory complaints.

In conclusion, the gastrointestinal complications of COVID-19 represent a complex and evolving aspect of the disease that warrants further exploration and understanding. The diverse range of manifestations, from acute pancreatitis to rectus sheath hematoma, emphasizes the importance of having a multidisciplinary approach to patient care that considers the potential gastrointestinal involvement of the virus [12]. By synthesizing the available literature on gastrointestinal complications of COVID-19, this systematic review aims to provide a comprehensive overview of the current understanding of these manifestations, their clinical implications, and the challenges they pose in the diagnosis and management of COVID-19 patients presenting with abdominal symptoms.

## 2. Methods

The protocol of this systematic review (not registered) was developed according to the preferred reporting items for systematic reviews and metanalysis (PRISMA) [13].

### 2.1. Information Sources and Search Strategy

This study is part of a project investigating the long-term and severe complications of COVID-19. An information professional conducted a comprehensive search that prioritized sensitivity to retrieve all relevant studies. The following databases were searched in October 2023: PubMed, Medline (Ovid, 1946–Current), Embase (Ovid, 1974–2021), Scopus, Web of Science, Science Direct, and Cochrane Library. The search was designed around keywords and controlled vocabulary that focused on “Long COVID” and variants (see Supplementary File S1 for full search details). No language or date restrictions were used. All database search results were imported into EndNote (version 19, Clarivate, Philadelphia, PA, USA) and exported to Covidence, where duplicates were removed prior to initial screening (Supplementary File S1).

### 2.2. Eligibility Criteria

No restrictions were made based on country, age, or gender. Any articles that did not have primary data, such as review articles, were excluded from the study after removing the duplicates. Furthermore, studies that were not in English were excluded. Only full articles were included, while any conference abstracts were excluded. During the full text screening, any studies that reported any disorder related to the GI tract (excluding liver) were included. We only included severe and/or long-term complications. The inclusion criteria related to this point included any patients who developed a related disorder after recovering from COVID-19. If the study did not mention clearly whether the disorder was diagnosed during the active infection or after recovery, the study was included if the GI disorder was diagnosed at least a month after COVID-19 diagnosis, or if the study reported that anti-SARS-CoV-2 IgG, but not IgM antibodies, was detected. The studies that reported any related diagnosis during the active COVID-19 infection were included only if the symptoms lasted for more than 12 weeks after COVID-19 diagnosis, if the GI disorder required surgical intervention, if treatment lasted for at least 12 weeks after COVID-19 diagnosis, or if the patient died before 12 weeks. Any cases with digestive tract disorders that were diagnosed during the active infection of COVID-19 and fully recovered within less than 12 weeks were excluded (Supplementary File S1).

### 2.3. Study Selection and Data Collection

Title and abstract screening, full-text screening, data extraction, and cross checking were conducted by two independent reviewers for each study using Covidence, and disagreements were resolved by consensus.

### 2.4. Data Items

Demographic and clinical data, including age, sex, comorbidities, treatments, and outcomes, were collected. Continuous variables were expressed as mean ± standard deviation or range of results. Categorical variables were expressed as percentages.

### 2.5. Risk of Bias and Quality Assessment

The quality of the included studies was assessed using different methods depending on the type of study. The Newcastle–Ottawa Quality Assessment Scale was used to assess the cohort studies [14]. The Jadad scale [15] was used to assess the randomized clinical trials, and the scale developed by Murad et al. was used to assess the case reports and case series [16]. Quality assessment was conducted by two independent reviewers.

### 2.6. Data Analysis

The digestive tract disorders reported by the included studies were classified under the following categories: gastrointestinal infections, inflammatory conditions, vascular disorders, structural abnormalities, and any other diagnosed abnormalities, in addition to any undiagnosed conditions.

## 3. Results

**Figure 1:**
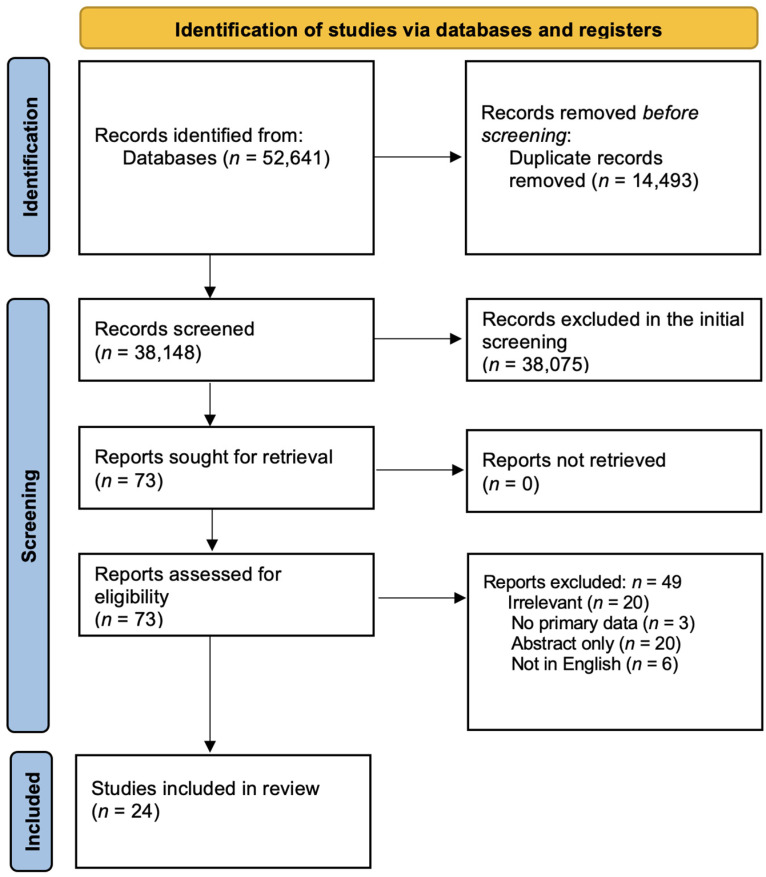
Screening and study-selection protocol.

Figure 1 shows the flow diagram of our protocol. After removing the duplicates, the titles and abstracts of 38,148 studies were screened, of which 73 were selected for full-text screening. Only 24 studies met our inclusion criteria. Of the 49 excluded studies, 20 were irrelevant, 3 had no primary data, 20 were conference abstracts, and 6 were not in English. Supplementary Table S1 summarizes the demographic and clinical data of the included subjects, as well as the quality-assessment score for each study [17,18,19,20,21,22,23,24,25,26,27,28,29,30,31,32,33,34,35,36,37,38,39,40,41].

### 3.1. Types of Studies and Demographic Data

Of the 24 included studies, 12 were case reports, 2 were randomized control studies, 6 were cohort studies, 3 were case-control studies, and 1 was a cross-sectional study. Among the 24 included studies, 2 were from Brazil, 1 from Egypt, 3 from India, 2 from Turkey, 1 from South Korea, 1 from Pakistan, 1 from Austria, 1 from the UK, 2 from Japan, 2 from the USA, 1 from Thailand, 1 from Saudi Arabia, and 1 from Italy (excluding 6 studies that did not report the country). The quality-assessment scores ranged from 4–6/6 for the case series and case reports (using Murad et al., scale, Table S2 [16]), 2–8/8 for the cohort studies (using NOS, Table S3 [14]) and 2–5/5 for clinical trials (using Jadad scale, Table S4 [15]).

The total number of patients reported by 12 case reports was 13 (9 M and 4 F), and their age ranges from 13 to 82 years. Xu et al. reported a large cohort of 154,068 patients, and the remaining 11 studies reported a total of 2283 patients who all had COVID-19. Many of these studies reported that more than 50% of the patients were male, and they were aged >18 years, with the highest reported mean age being 61.42 ± 15.64. Of these, 862 developed at least one type of GI disorder. All these patients had at least one type of GI disorder during active COVID-19 infection or developed at least one type of GI disorder after recovering from COVID-19. This disorder was either fatal, severe, required surgical intervention, or lasted (or required treatment) for more than 12 weeks. The total number of control patients with no history of COVID-19 was 975, in addition to a total of 11,498,416 control patients who were reported by Xu et al. [41].

### 3.2. Clinical Data

**Figure 2:**
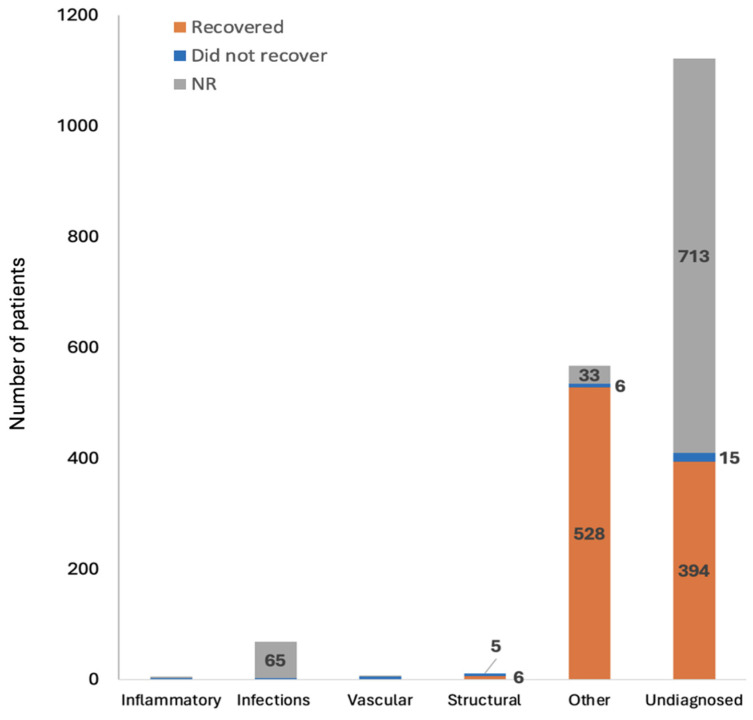
Types of GI tract (except liver) injury/disorders post-COVID-19 infection as reported by 24 studies and their outcomes. The number on each segment shows the number of patients. The inflammatory disorders included 5 patients, of which 1 recovered, 2 did not recover, and the outcome was not reported for the other 2 patients. Disorders with GI infections included 68 patients, of which 1 recovered, 2 did not recover, and the outcome was not reported for 65 patients. Disorders caused by vascular problems included 7 patients, of which 1 recovered, 4 did not recover, and the outcome was not reported for 2 patients. NR: Not reported.

The data across our study encompass a wide range of disorders categorized into inflammatory conditions, gastrointestinal infections, vascular disorders, structural abnormalities, other diagnosed abnormalities, and undiagnosed conditions. Each table details specific conditions such as ulcerative colitis (UC), mesenteric thrombosis, nausea, and dysphagia, with outcomes varying from recovery to non-recovery or not reported (NR) (Tables S5–S10). Figure 2 also outlines the number of patients in each category. Several studies overlap in their examination of multiple disorders, resulting in some studies and patients being counted across different categories.

#### 3.2.1. Post-COVID-19 GI Inflammatory Conditions

**Figure 3:**
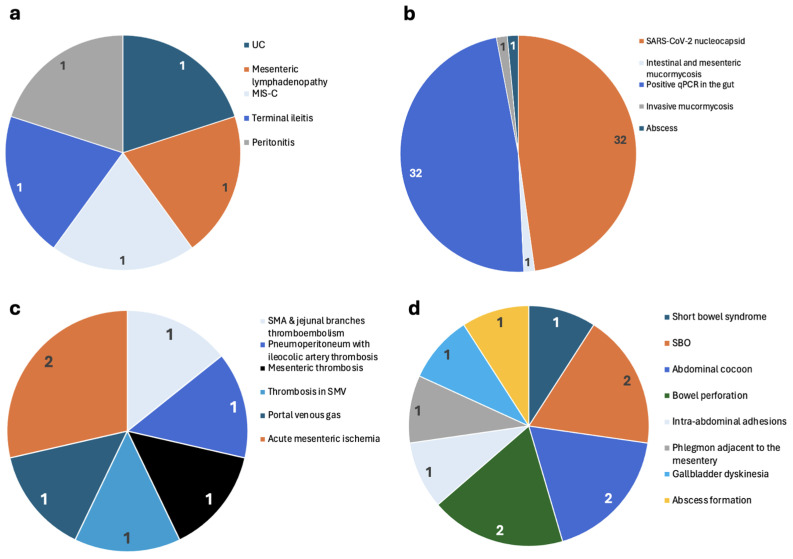
Types of diagnosed GI tract (except liver) injury/disorders post-COVID-19 infection, including inflammatory (**a**), infections (**b**), vascular (**c**), and structural (**d**). The number in each section shows the number of patients. (**a**): Inflammatory disorders, as reported by five studies. Numbers for acute hepatitis and acute gastritis were NR. (**b**): Disorders caused by GI infections as reported by five studies. Numbers for *Cryptosporidium* spp. and *H. pylori* infections were NR. (**c**): GI vascular disorders, as reported by six studies. (**d**): GI structural disorders, as reported by seven studies. NR: Not reported. UC: Ulcerative colitis; MIS-C: multisystem inflammatory syndrome in children; qPCR: quantitative polymerase chain reaction; SMA: superior mesenteric artery; SMV: superior mesenteric vein; SBO: small bowel obstruction.

Table S5 summarizes the reported post-COVID-19 GI inflammatory conditions. Figure 3a also outlines the observed inflammatory conditions, along with the number of patients involved. One study reported that a patient with UC recovered successfully [35]. The same study mentioned mesenteric lymphadenopathy but did not specify the outcome [35]. Acute pancreatitis was reported in two studies, but neither provided specific patient numbers nor outcomes [38,42]. A single study on acute gastritis did not report patient numbers or outcomes [41]. Multisystem Inflammatory Syndrome in Children (MIS-C), terminal ileitis [38], and peritonitis [40] were each reported once. Similarly, in total, excluding patients from studies by Marinoni and Xu, there were five reported GI patients [37,41]. The outcomes of the reported disorders are summerized in Table S5.

#### 3.2.2. Post-COVID-19 GI Infections

Table S6 summarizes the reported post-COVID-19 GI infections and their outcomes. Figure 3b also outlines the observed infectious conditions, along with the number of patients involved. A study by Enas et al. (2023) on *Cryptosporidium* spp. and *H. pylori* infection did not specify the number of patients or their outcomes [19]. Zollner et al. (2022) detected SARS-CoV-2 anti-nucleocapsid in the intestines of 32 out of 46 patients [21], while Banerjee et al. (2023) reported intestinal and mesenteric mucormycosis in one patient [25]. Another study by Zollner et al. (2022) found a positive qPCR signal in at least one segment of the gut in 32 out of 46 patients [21]. Jain et al. (2022) studied invasive mucormycosis in one instance, resulting in one patient not recovering [27].

Additionally, Tong et al. (2022) reported phlegmon adjacent to the mesentery in one patient, who did not recover, while Gundogdu et al. (2022) reported abscess formation in one patient, who recovered [33,38]. Excluding patients from the study by Enas et al., the total number of gastrointestinal patients reported across these conditions was 68 [19]. Among these patients, specific recovery outcomes were reported for one patient, while the outcomes for 65 patients were not disclosed.

#### 3.2.3. Post-COVID-19 GI Vascular Disorders

Table S7 and Figure 3c summarize the reported post-COVID-19 GI vascular disorders and the number of patients involved and their outcomes. Parrela et al. (2022) reported a case of superior mesenteric artery (SMA) and jejunal branches thromboembolism, where the patient did not recover [18]. Banerjee et al. (2023) reported a case of pneumoperitoneum with thrombosis of the ileocolic artery [24], while Jain et al. (2022) reported mesenteric thrombosis in one patient, who did not recover [27]. Gundogdu et al. (2022) studied thrombosis in the superior mesenteric vein (SMV), with the patient recovering [33].

Another study by Hosoda et al. (2022) found portal venous gas in one patient, who did not recover [39]. Acute mesenteric ischemia was covered in two studies: one by Hosoda et al. (2022) and another by Basravi et al. (2023) [40,41]. Among a total of seven gastrointestinal patients studied across these conditions, one patient was reported as recovered.

#### 3.2.4. Post-COVID-19 GI Structural Abnormalities

Table S8 summarizes the reported post-COVID-19 GI structural abnormalities and their outcomes. Figure 3d also outlines the observed structural pathologies along with the number of patients involved. Parrela et al. (2022) reported a case of short bowel syndrome, with the patient not recovering [18]. AbdurRaheem et al. (2022) and Basravi et al. (2023) conducted studies on SBO, with a total of three patients across both studies [26,40]. One patient recovered according to Basravi et al. (2023) [40]. AbdurRaheem et al. (2022) also studied abdominal cocoons, with both patients reported to have recovered [26].

Bowel perforation was examined in studies by Abbassi et al. (2021) and Gundogdu et al. (2022), with one patient not recovering in Abbassi et al.’s (2021) study and one recovering in Gundogdu et al.’s (2022) study [33,40]. Intra-abdominal adhesions were reported by Abbassi et al. (2021), with the patient not recovering [31]. Phlegmon adjacent to the mesentery was studied by Tong et al. (2022), with the patient not recovering [38]. Wang et al. (2022) reported on gallbladder dyskinesia, with the patient recovering [30]. Gundogdu et al. (2022) also reported on abscess formation, with the patient recovering [33]. In total, across these studies, there were 11 GI patients reported, with 6 recovering and 5 not recovering.

#### 3.2.5. Other Post-COVID-19 GI Diagnosed Abnormalities

**Figure 4:**
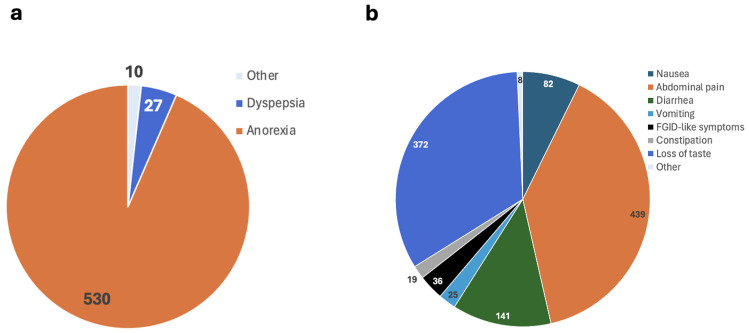
Other diagnosed or undiagnosed post-COVID-19 GI disorders. The number in each section shows the number of patients. (**a**): Dyspepsia, anorexia, and other diagnosed GI disorders reported post-COVID-19 infection, as reported by 13 studies. There are 10 patients in Figure 4a who were diagnosed with GERD, SIBO, gastric ulcers, duodenal ulcers, hematochezia, erosions and luminal bleeding of colon, ascites, extensive pneumatosis in gastric intestinalis, hemorrhagic necrosis in gastric and extensive small bowel mucosa, and esophageal dysphagia. Also, the number of patients who developed IBS post-COVID-19 was reported as 25%, and the number of patients who developed PUD was NR. (**b**): Reported symptoms/undiagnosed GI disorders post-COVID-19 infection. NR: Not reported; GERD: gastroesophageal reflux disease; SIBO: small intestinal bacterial overgrowth; PUD: peptic ulcer disease.

Table S9 and Figure 4a summarize the other reported post-COVID-19 GI-diagnosed abnormalities that do not fall under the above categories, along with the number of patients involved. Lee et al. (2021) reported oropharyngeal dysphagia in one patient [23]. Dyspepsia was studied in three separate studies: Anayat et al. (2022), Cooney et al. (2022), and Xu et al. (2023) [25,28,41]. Cooney et al. (2022) reported outcomes for 67% of 30 patients, with 27 patients not recovering out of 122 total [28]. Anorexia was examined in studies by Sandal et al. (2023) and Chancharoenthana et al. (2023), with 530 out of 585 patients not recovering and 525 patients recovering [29,34]. Gastroesophageal Reflux Disease (GERD) was studied in three instances by Wang et al. (2022), Marinoni et al. (2023), and Xu et al. (2023), with one patient reported to have recovered [30,37,41]. Small intestinal bacterial overgrowth (SIBO) was reported by Wang et al. (2022), with one patient recovering [30].

Abbassi et al. (2021) reported on gastric ulcers and duodenal ulcers, with both patients not recovering [31]. Morita et al. (2023) studied hematochezia and erosions with luminal bleeding of the colon, both resulting in the patient not recovering [35]. Altuwaijri et al. (2023) and Xu et al. (2023) reported on irritable bowel syndrome (IBS) [36,41]. Hosoda et al. (2022) reported ascites, extensive pneumatosis in gastric intestinalis, and hemorrhagic necrosis in gastric and extensive small bowel mucosa, all resulting in patients not recovering [39], while Xu et al. (2023) also studied peptic ulcer disease (PUD) [41]. In total, excluding patients from the studies by Altuwaijri and Xu, there were 567 GI patients studied across these conditions [36,41]. Outcomes indicated that 528 patients recovered, while outcomes for 33 patients were not reported, and 6 patients did not recover.

#### 3.2.6. Post-COVID-19 GI Undiagnosed Conditions

Table S10 summarizes the reported undiagnosed post-COVID-19 GI conditions and their outcomes. Figure 4b also outlines the observed undiagnosed conditions, along with the number of patients involved. Parrela et al. (2022), Golla et al. (2023), Banerjee et al. (2023), AbdurRaheem et al. (2022), Cooney et al. (2022), Wang et al. (2022), Abbassi et al. (2021), Natarajan et al. (2022), Gundogdu et al. (2022), Altuwaijri et al. (2023), and Marinoni et al. (2023) collectively reported on nausea across 11 studies, involving 561 patients, with 2 patients not recovering and 2 recovering [18,20,24,26,28,30,31,32,33,36,37]. For abdominal pain, reported in 17 studies by Parrela et al. (2022), Golla et al. (2023), Banerjee et al. (2023), AbdurRaheem et al. (2022), Jain et al. (2022), Cooney et al. (2022), Sandal et al. (2023), Wang et al. (2022), Abbassi et al. (2021), Natarajan et al. (2022), Gundogdu et al. (2022), Chancharoenthana et al. (2023), Morita et al. (2023), Altuwaijri et al. (2023), Marinoni et al. (2023), Tong et al. (2022), and Xu et al. (2023), a total of 1149 patients were examined, with 5 not recovering and 16 recovering [18,20,24,26,27,28,29,30,31,32,33,34,35,36,37,38,41]. Diarrhea, examined in 10 studies by Parrela et al. (2022), Enas et al. (2023), Golla et al. (2023), Ferreira et al. (2022), Cooney et al. (2022), Natarajan et al. (2022), Morita et al. (2023), Altuwaijri et al. (2023), Marinoni et al. (2023), and Xu et al. (2023), involved 705 patients, with 2 not recovering [18,19,20,22,28,32,35,36,37,41]. Hyporexia, reported in three studies by Parrela et al. (2022), Wang et al. (2022), and Marinoni et al. (2023), had two patients, with one not recovering [18,30,37].

Vomiting, examined in 12 studies by Parrela et al. (2022), Enas et al. (2023), Golla et al. (2023), Banerjee et al. (2023), AbdurRaheem et al. (2022), Sandal et al. (2023), Abbassi et al. (2021), Natarajan et al. (2022), Gundogdu et al. (2022), Altuwaijri et al. (2023), Marinoni et al. (2023), and Xu et al. (2023), involved 446 patients, with 2 not recovering and 3 recovering [18,19,20,24,26,29,31,32,33,36,37,41]. Weight loss, examined in one study by Enas et al. (2023), and functional GI disorder (FGID)-like symptoms, reported in 1 study by Golla et al. (2023), had 127 and 320 patients, respectively [19,20]. Dysphagia, reported in one study by Lee et al. (2021), involved one patient [24]. Constipation, examined in 6 studies by AbdurRaheem et al. (2022), Jain et al. (2022), Cooney et al. (2022), Wang et al. (2022), Hosoda et al. (2022), and Xu et al. (2023), was reported in 19 patients out of a collective number of 127 patients with gastrointestinal complications, with 1 patient recovering and 2 not recovering [26,27,28,30,39,41].

## 4. Discussion

The aim of our study is to systematically review and analyze the prevalence, risk factors, clinical manifestations, and potential mechanisms of GI complications (excluding liver complications, which are studied separately) in patients post-COVID-19 infection. This review provides insight into the spectrum of GI issues such as infections, inflammatory conditions, vascular disorders, and structural abnormalities that may arise following COVID-19 recovery. By synthesizing the available data, we aimed to contribute to a better understanding of the long-term effects of COVID-19 on the GI system and guide healthcare professionals in managing and monitoring these complications effectively.

Our systematic review included 24 studies. Post-COVID-19 infection, GI complications encompass a spectrum of conditions, which we categorized into six broad groups. These include GI infections, inflammatory conditions, vascular disorders, structural abnormalities, other diagnosed abnormalities, and undiagnosed conditions. GI infections range from microbial invasions like *Cryptosporidium* spp. and *H. pylori* infections to the detection of SARS-CoV-2 anti-nucleocapsid in intestines, with the highest prevalence of a complication in the infections category seen in the latter. Inflammatory responses within the GI tract manifest as conditions like UC and acute pancreatitis, with acute gastritis showing the highest prevalence. Vascular complications impact blood flow to the GI system, resulting in conditions such as thromboembolism and mesenteric thrombosis, with superior mesenteric artery thromboembolism showing notable prevalence.

Structural abnormalities post-COVID-19 infection, such as SBO, often lead to complications like bowel perforation, with SBO exhibiting the highest prevalence. Other diagnosed abnormalities include various GI issues beyond infections and inflammation, with dyspepsia showing the highest prevalence. Undiagnosed conditions post-COVID-19 infection present as symptoms like nausea and abdominal pain, with abdominal pain demonstrating the highest prevalence, highlighting its significant occurrence as a symptom post-COVID-19 infection.

### 4.1. Post-COVID-19 GI Inflammatory Conditions

Inflammatory conditions post-COVID-19 infection have been a significant concern, with various studies reporting complications such as UC, mesenteric lymphadenopathy, and acute pancreatitis. These conditions have been linked to the body’s inflammatory response to the virus, often exacerbating pre-existing GI issues or triggering new inflammatory pathways. For instance, UC and mesenteric lymphadenopathy were documented in COVID-19 patients by Morita et al. (2023), highlighting the virus’s potential to aggravate chronic inflammatory diseases [35]. Acute pancreatitis was reported by Marinoni et al. (2023) and Xu et al. (2023), underscoring the need for vigilant monitoring of pancreatic health in affected individuals [37,41]. Additionally, cases of acute gastritis (Xu, 2023) and pediatric inflammatory multisystem syndrome (PIMS) (Tong et al., 2022) were noted, with some patients not recovering, indicating the severe impact of COVID-19 on the GI system [38].

COVID-19 has been associated with various GI inflammatory conditions, with several proposed mechanisms contributing to these manifestations. Impairments in the barrier function of the GI tract, GI inflammation, alterations in the gut microbiota, thromboembolic events, and changes in serotonin metabolism are suggested mechanisms, leading to GI symptoms in COVID-19 patients [40]. Additionally, studies have shown that SARS-CoV-2 infection may trigger the de novo occurrence of UC by affecting intestinal barrier function, activating T cells with a skewed T cell receptor repertoire, and increasing levels of anti-SARS-CoV-2 spike IgG antibodies [36]. Furthermore, alterations in the composition of the intestinal microbiota have been reported in individuals with COVID-19, which could contribute to the development of GI inflammatory conditions [37].

Moreover, beyond the acute phase of infection, individuals with COVID-19 have been found to have increased risks and burdens of incident GI disorders [41]. These long-term GI outcomes of COVID-19 suggest a complex interplay between the virus and the GI system, potentially leading to persistent inflammatory conditions. Additionally, severe cases of COVID-19, especially in children, have been associated with a hyperinflammatory response known as Pediatric Inflammatory Multisystem Syndrome Temporally associated with SARS-CoV-2 (PIMS-TS), which can involve GI manifestations, such as terminal ileitis, requiring surgical intervention [38].

Morita et al. (2023) observed a case of UC and mesenteric lymphadenopathy in a single patient who recovered following steroid therapy [35]. Acute pancreatitis was discussed in studies by Marinoni al. (2023) and Xu et al. (2023), though specific recovery or death rates were not reported [37,41]. Tong et al. (2022) documented PIMS and terminal ileitis, with one patient not recovering [38]. Basravi et al. (2023) reported a case of peritonitis in which the outcome was not recorded [40]. The severity ranged from moderate to severe, with management involving pharmacological treatments and supportive care. The studies highlight variable recovery outcomes, with some patients responding well to treatment, while others experienced severe and life-threatening complications.

### 4.2. Post-COVID-19 GI Infections

GI infections in post-COVID-19 patients have been documented, ranging from *Cryptosporidium* spp. and *H. pylori* infections to mucormycosis. These infections may be secondary to the immunosuppression or microbiome alterations caused by the virus. Enas et al. (2023) identified *Cryptosporidium* spp. and *H. pylori* infections in a study involving 2825 patients, although specific outcomes were not reported [19]. Zollner et al. (2022) detected SARS-CoV-2 anti-nucleocapsid in the intestines and positive qPCR signals in gut segments, indicating viral persistence in the GI tract [21]. The occurrence of mucormycosis, particularly intestinal and mesenteric mucormycosis, was identified in severely affected patients by Banerjee et al. (2023) and Jain et al. (2022), often with fatal outcomes [24,27]. This highlights the severe impact of opportunistic infections in immunocompromised post-COVID-19 patients and underscores the necessity for comprehensive care strategies to manage these infections effectively.

COVID-19 infections have been associated with various GI complications, which can manifest in different ways. One possible mechanism is the persistence of viral antigens in the gut mucosa even after the acute phase of COVID-19. Zollner et al. (2022) found that in patients with inflammatory bowel diseases (IBD), viral antigens from COVID-19 can persist in the gut mucosa, indicating a potential link between COVID-19 and ongoing GI issues [21]. This persistence of viral antigens may trigger inflammatory responses or disrupt the normal gut microbiota, leading to GI complications.

Moreover, COVID-19 can also predispose individuals to secondary infections such as mucormycosis, which can affect the intestines and mesentery. Banerjee et al. (2021) reported a case of intestinal and mesenteric mucormycosis in a patient with uncontrolled diabetes following a recent COVID-19 infection [24]. The immune dysregulation caused by COVID-19 may create a favorable environment for opportunistic infections like mucormycosis to take hold in the GI tract, resulting in severe complications such as bowel ischemia and gangrene.

Additionally, Jain et al. (2021) highlighted the occurrence of GI invasive mucormycosis in post-COVID-19 patients, emphasizing the importance of considering this fungal infection in individuals presenting with mesenteric ischemia or bowel perforation after recovering from COVID-19 [27]. The challenges in diagnosing such secondary infections post-COVID-19 underscore the need for a high index of suspicion and vigilance in managing GI complications in these patients.

The study by Enas et al. (2023) reported infections with *Cryptosporidium* spp. and *H. pylori* in COVID-19 patients but did not provide recovery or death rates [19]. Zollner et al. (2022) found SARS-CoV-2 anti-nucleocapsid proteins in the intestines of 34 out of 76 patients, though the study did not detail recovery outcomes [21]. Banerjee et al. (2023) and Jain et al. (2022) documented cases of intestinal and mesenteric mucormycosis, with one patient recovering and one not [24,27]. The study by Gundogdu et al. (2022) reported an abscess formation in one patient who eventually recovered [33]. The severity of infections varied from mild to severe, with management strategies including surgical interventions such as thromboembolectomy and abscess drainage, and pharmacological treatments like antifungal therapy. Recovery rates were limited to specific cases, and death rates were noted in some studies due to severe complications like invasive mucormycosis.

### 4.3. Post-COVID-19 GI Vascular Disorders

Vascular complications post-COVID-19 are alarming due to their potential severity, including thromboembolism, mesenteric thrombosis, and acute mesenteric ischemia. Thromboembolism in the SMA and jejunal branches was reported by Parrela et al. (2022) in COVID-19 patients with fatal outcomes, indicating a severe thrombotic risk associated with the virus [18]. Mesenteric thrombosis and portal venous gas were also documented by Jain et al. (2022) and Hosoda et al. (2022), respectively, with some patients not recovering [27,39]. Acute mesenteric ischemia was noted in several cases by Hosoda et al. (2022) and Basravi et al. (2023), with mixed recovery outcomes, emphasizing the critical nature of these vascular disorders [39,40]. These findings highlight the need for early detection and intervention to manage vascular complications effectively in post-COVID-19 patients.

COVID-19 infection has been associated with various GI vascular complications, including mesenteric ischemia, mesenteric thrombosis, intestinal perforation, and GI necrosis. These complications can arise due to the virus’s impact on the vascular system, leading to thrombotic events and subsequent ischemic damage to the GI tract. Patients with COVID-19 may develop mesenteric ischemia even in the absence of traditional risk factors, highlighting the direct influence of the virus on vascular health [40]. Additionally, post-COVID-19 syndrome can manifest as mesenteric thrombosis, necessitating close monitoring of patients for the early detection of such serious complications [33].

Furthermore, COVID-19 has been linked to rare but severe conditions like GI invasive mucormycosis, which can present as mesenteric ischemia or bowel perforation in post-COVID-19 patients [27]. The development of portal vein thrombosis (PVT) has also been reported as a rare thrombotic complication in COVID-19, contributing to extensive GI necrosis in some cases [39]. These findings underscore the diverse ways in which COVID-19 can impact the GI vasculature, leading to potentially life-threatening consequences.

Parrela et al. (2022) reported a case of SMA and jejunal branches thromboembolism, resulting in non-recovery [18]. Banerjee et al. (2023) and Jain et al. (2022) noted cases of thrombosis, with mixed outcomes: one patient recovered, and the other did not [24,27]. Hosoda et al. (2022) described a case of portal venous gas and acute mesenteric ischemia, leading to death [40]. The management of these conditions typically involved anticoagulation therapy and surgical interventions. The severity of these vascular disorders was generally high, reflecting significant morbidity and mortality rates in affected patients. The recovery rates were low, with several patients experiencing fatal outcomes due to severe vascular complications.

### 4.4. Post-COVID-19 GI Structural Abnormalities

Post-COVID-19 structural abnormalities in the GI tract include conditions like short bowel syndrome, SBO, and abdominal cocoon. Short bowel syndrome was observed by Parrela et al. (2022), with the patient not recovering [18]. SBO was reported in studies by AbdurRaheem et al. (2022) and Basravi et al. (2023), with mixed recovery outcomes [26,40]. Bowel perforation and intra-abdominal adhesions were also reported by Abbassi et al. (2021) and Gundogdu et al. (2022), often with severe consequences [31,33]. The occurrence of conditions, such as phlegmon adjacent to the mesentery [38] and gallbladder dyskinesia [30], further illustrates the diverse structural complications that can arise post-COVID-19, necessitating targeted therapeutic interventions to manage these conditions.

COVID-19 infection has been associated with various complications in the GI tract, including perforation, SBO, short bowel syndrome, adhesions, abscesses, and phlegmon formation. These complications can arise due to several mechanisms related to the impact of the SARS-CoV-2 virus on the GI system. One significant complication is intestinal perforation, which can occur as a result of mesenteric thrombosis associated with COVID-19. The virus’s interaction with angiotensin-converting enzyme 2 (ACE2) receptors in the GI tract can lead to mesenteric ischemia, compromising blood flow and potentially resulting in perforation and abscess formation [33].

Moreover, COVID-19 infection has been linked to small bowel obstruction and short bowel syndrome, conditions that can develop due to post-inflammatory strictures or adhesions in the intestines following severe infection. The inflammatory response triggered by the virus can lead to scarring and narrowing of the bowel lumen, causing obstructions and potentially resulting in short bowel syndrome, where there is inadequate absorption of nutrients and fluids due to reduced bowel length [38].

In addition to these complications, COVID-19 has been associated with the formation of abscesses and phlegmons in the GI tract. The presence of microthrombi in the GI tract following COVID-19 infection can lead to chronic nonhealing ulcers, recurrent bleeding, and subsequent perforations with fistulization, potentially resulting in abscess formation. The development of phlegmons, which are localized areas of inflammation and infection, may also occur as a consequence of the virus-induced inflammatory processes in the GI system [31].

Parrela et al. (2022) reported a case of short bowel syndrome with no recovery [18]. Studies by AbdurRaheem et al. (2022) and Basravi et al. (2023) described small bowel obstruction, with mixed outcomes: one patient recovered, while another did not [26,40]. AbdurRaheem et al. (2022) also reported two cases of abdominal cocoon, both of which recovered [26]. Abbassi et al. (2021) and Gundogdu et al. (2022) noted cases of bowel perforation, with one patient recovering and another not [31,33]. The management of these conditions involved surgical interventions such as resections and anastomoses. The severity ranged from moderate to severe, with significant morbidity associated with these structural complications. Recovery was variable, with some patients responding well to surgical management, while others faced fatal outcomes.

Overall, the structural abnormalities and complications observed in the GI tract of COVID-19 patients, such as perforation, small bowel obstruction, short bowel syndrome, adhesions, abscesses, and phlegmon formation, are multifactorial and can be attributed to the direct effects of the virus on intestinal cells, the inflammatory response, and the potential long-term consequences of the infection.

### 4.5. Other Post-COVID-19 GI Diagnosed Abnormalities

Other diagnosed GI abnormalities include conditions such as dyspepsia, anorexia, GERD, and various types of ulcers. Dyspepsia was frequently reported by Anayat et al. (2022), Cooney et al. (2022), and Xu et al. (2023), affecting a significant number of patients, with varying recovery outcomes [25,28,41]. Anorexia was documented by Sandal et al. (2023) and Chancharoenthana et al. (2023), with a majority of patients recovering [29,34]. GERD and small intestinal bacterial overgrowth (SIBO) were noted by Wang et al. (2022) and Xu et al. (2023), all of whom recovered, indicating a generally good prognosis for these conditions post-COVID-19 [30,41]. However, gastric and duodenal ulcers were associated with more severe outcomes, with some patients not recovering, as reported by Abbassi et al. (2021) [31]. These findings underscore the importance of monitoring and managing a wide range of GI abnormalities in post-COVID-19 care.

These complications can be attributed to various mechanisms resulting from the impact of the virus on the GI system. The presence of angiotensin-converting enzyme 2 (ACE2) receptors throughout the GI tract has been implicated in the pathophysiology of GI manifestations in COVID-19 [28]. This widespread distribution of ACE2 receptors facilitates viral entry and subsequent damage to the GI mucosa, contributing to the development of complications.

Furthermore, COVID-19 infection has been linked to subacute enteritis characterized by mucosal bleeding, perforation, and internal fistulas in the GI tract [31]. The formation of chronic non-healing ulcers due to concomitant microthrombi in the GI tract can result in recurrent bleeding, perforations, and fistulization, leading to severe complications such as extensive pneumatosis in gastric intestinalis and hemorrhagic necrosis in the gastric and small bowel mucosa. The presence of the SARS-CoV-2 virus in the GI tract can trigger inflammatory responses and disrupt the integrity of the GI barrier, contributing to the development of conditions like UC [35]. Additionally, COVID-19 has been associated with portal and mesenteric vein thrombosis, which can result in extensive GI necrosis and further exacerbate complications in the post-acute phase of the infection [39].

Moreover, the dysregulation of the gut microbiota following COVID-19 infection can play a role in the pathogenesis of GI symptoms. Nutritional modulation of the gut microbiota has been shown to alleviate severe GI symptoms in patients with post-acute COVID-19 syndrome, highlighting the importance of the gut microbiome in maintaining GI health during and after the infection [30]. Patients with pre-existing conditions such as Parkinson’s disease may experience prolonged dysphagia following COVID-19 infection, indicating a potential exacerbation of underlying GI issues in vulnerable populations [23]. The interplay between viral-induced inflammation, immune responses, and alterations in the gut microbiota likely contributes to the diverse range of GI complications observed in COVID-19 patients.

Lee et al. (2021) documented a case of oropharyngeal dysphagia, with no recovery details provided [23]. Dyspepsia was reported by Anayat et al. (2022), Cooney et al. (2022), and Xu et al. (2023), though recovery outcomes were not specified [25,28,41]. Studies on anorexia by Sandal et al. (2023) and Chancharoenthana et al. (2023) showed high recovery rates, with most patients improving [29,34]. Wang et al. (2022) reported cases of GERD and SIBO, with the patient recovering [30]. Severe cases of gastric and duodenal ulcers documented by Abbassi et al. (2021) did not result in recovery [31]. Morita et al. (2023) noted hematochezia, with no recovery reported [35]. Management varied, including dietary changes, medications, and surgical interventions. The severity of these conditions was generally moderate, with variable recovery rates and some conditions leading to severe complications.

### 4.6. Post-COVID-19 GI Undiagnosed Conditions

Undiagnosed GI symptoms post-COVID-19, such as nausea, abdominal pain, diarrhea, and vomiting, present a significant challenge in clinical management. Nausea was reported in 11 studies, with varying outcomes. Abdominal pain was the most frequently reported symptom in 17 studies, affecting 439 out of 1149 patients, with some not recovering. Diarrhea and vomiting were also common, with mixed recovery outcomes, as noted in studies by Parrela et al. (2022), Enas et al. (2023), Golla et al. (2023), Banerjee et al. (2023), and others [18,19,20,24]. Additionally, weight loss and functional GI disorder (FGID)-like symptoms were less frequently reported but still significant, requiring further investigation to understand their pathophysiology post-COVID-19. These findings emphasize the need for comprehensive symptom management and further research to address the chronic GI issues that can persist following COVID-19 infection.

Undiagnosed conditions observed include nausea, abdominal pain, diarrhea, hyporexia, vomiting, weight loss, FGID-like symptoms, dysphagia, constipation, regurgitation, loss of taste, and abdominal distension/bloating. Studies such as those by Parrela et al. (2022), Golla et al. (2023), Banerjee et al. (2023), and others reported a range of symptoms with mixed outcomes [18,20,24]. While many patients experienced persistent symptoms, several studies noted recovery over time. For instance, Chancharoenthana et al. (2023) reported high recovery rates for loss of taste [34]. Management often involved supportive care and symptom-specific treatments. The severity of undiagnosed conditions varied widely, with some patients experiencing mild symptoms and others facing more severe, persistent issues. Recovery rates were generally positive, although the lack of specific data in some studies makes comprehensive conclusions difficult.

### 4.7. Proposed Mechanisms for Development of COVID-19-Related GI Complications

COVID-19 infection can lead to a spectrum of GI complications through various mechanisms involving viral entry, inflammation, thrombosis, microbiota dysregulation, and immune responses. Understanding these mechanisms is crucial for the management and treatment of COVID-19 patients with GI manifestations, as these complications can have long-lasting effects on the health and well-being of individuals post-infection. The proposed mechanisms of developing gastrointestinal (GI) disorders following COVID-19 involve multiple pathways, including direct viral invasion, immune-mediated injury, and alterations in gut microbiota.

SARS-CoV-2, the virus responsible for COVID-19, utilizes the ACE2 receptors to enter host cells. These receptors are abundantly expressed in the gastrointestinal tract, allowing the virus to directly infect the epithelial cells lining the gut. This viral invasion can lead to direct cytopathic effects, causing inflammation and damage to the GI mucosa [42]. Additionally, the immune response triggered by the infection can result in widespread inflammation, contributing to GI symptoms such as diarrhea, nausea, and abdominal pain. The cytokine storm, characterized by elevated levels of pro-inflammatory cytokines, further exacerbates mucosal damage and disrupts the gut-barrier function [43].

COVID-19 can induce significant alterations in the gut microbiota, a critical component of the gastrointestinal system’s health and function. Dysbiosis, or the imbalance of gut microbiota, has been observed in COVID-19 patients, potentially due to the infection itself or the use of antibiotics and other treatments [44]. It was reported that fecal RNA was detected in more than 50% of COVID-19 patients with a fecal viral shedding duration of 1–33 days after symptomatic recovery of lung injury and even after a negative nasopharyngeal swab [45,46]. Furthermore, Zuo et al. reported that the opportunistic pathogens and commensals were depleted in 15 COVID-19 patients. They also reported that *Coprobacillus*, *Clostridium ramosum*, and *Clostridum mathewayi* were more commonly detected in patients with severe COVID-19, while *Faecalibacterium prausnitzii* was detected in milder cases [44]. This imbalance can lead to impaired immune regulation and increased susceptibility to secondary infections like mucormycosis, and the potential for post-COVID-19 complications such as cryptosporidiosis.

In addition to secondary infections, COVID-19 can lead to a range of GI inflammatory conditions through various mechanisms, including disruptions in the gut-barrier function, alterations in the gut microbiota, immune dysregulation, and hyperinflammatory responses. One explanation for the hyperinflammatory response could be the vital role of ACE2 in regulating the Renin-angiotensin system (RAS). Angiotensinogen (Ang) is converted to Ang I by renin, and then to Ang II by ACE, primarily in the lungs. The binding of Ang II to Ang T1 receptors (AT1R) on affected cell membranes leads to a pro-inflammatory, pro-fibrotic, and vasoconstrictive state. A tight physiological balance exists to counterbalance the undesired adverse effect of Ang II. ACE2 converts Ang II to Ang 1–7, which exerts opposing effects on Ang II, including vasodilation, anti-inflammation, and anti-fibrosis. Also, ACE2 converts Ang I to Ang 1–9, which is then converted to Ang 1–7 by ACE. A third pathway to generate Ang 1–7 involves neprilysin (neural endopeptidase-NEP), which converts Ang I to Ang 1–7. There is a balance between Ang II and And 1–7 to maintain vascular homeostasis and reduce excessive inflammation and remodeling. SARS-CoV-2 disrupts this balance while invading the host cell. The attachment of the virus is facilitated by the modification and cleavage of viral spike proteins by convertases such as transmembrane protease serine (TMPRSS 2), which assists in the internalization and replication of the virus in the host cell. The binding and downregulation of ACE2 shifts the RAS pathway towards a pro-inflammatory and pro-thrombotic state, exacerbating endothelial damage and dysfunction. Inflammation by unopposed Ang II actions activates ADAM 17, which depletes cell-bound ACE2 and Ang 1–7 synthesis, increasing cytokine production like TNF-α. This unopposed effect causes an increase in inflammation, hypercoagulation, and fibrosis [47].

Additionally, unopposed Ang II activity due to SARS-CoV-2 increases the risk of thrombosis. Ang II at AT1R, enhances tissue factor (TF) expression on endothelial cells, initiating a clotting cascade. This contributes to the hypercoagulable state seen in COVID-19 patients, where blood clots can form in small and large vessels. This may explain the reported GI vascular complications, ranging from mesenteric ischemia to vascular damage. Moreover, SARS-CoV-2 can trigger coagulopathy through NETs (neutrophil extracellular traps). When recruited, neutrophils can also undergo a process called NETosis, where they release NETs—a web-like structure composed of DNA, histones, and various microbicidal proteins. NETs contribute to coagulation and inflammation in COVID-19 patients by increasing TF expression and platelet aggregation. NETs also stimulate complement activation, further exacerbating inflammation and thrombosis, with C3 and C5 complement components having a crucial role in hypercoagulation. Studies have shown that inhibiting these complement components reduces inflammation and NET release in COVID-19 patients. SARS-CoV-2 can activate the complement system through the classic, alternative, and lectin pathways. Along with NET formation, the complement activity contributes to a feedback loop that worsens coagulopathy and tissue damage. Additionally, platelet dysfunction has a significant role in COVID-19-associated coagulopathy (CAC). Complement pathways, inflammatory cytokines, anti-SARS-CoV-2 immunoglobulins, and other mediators heighten platelet activation. Activated platelets have a role in immunothrombosis as they release pro-thrombotic factors and promote platelet-neutrophil aggregation, thus releasing NETs and enhancing inflammation and thrombosis [48].

In summary, the interplay between the RAS imbalance, NETs, complement activation, and platelet dysfunction in COVID-19 leads to a complex network of hyperinflammation and coagulation that causes the severe clinical presentation of this disease.

### 4.8. Study Limitations

This systematic review has several limitations that should be considered when interpreting the findings. Firstly, there was significant overlap between studies in reporting multiple gastrointestinal disorders, often within the same patient cohort. This overlap can complicate the categorization and analysis of specific complications, potentially leading to an overestimation or underestimation of certain disorders. Another limitation is the small number of included studies. To maintain high quality and avoid duplication, we excluded conference abstracts, which limited the pool of available data. This exclusion criterion aimed to ensure that the work was not published both in a conference proceeding and a journal, thereby avoiding redundancy. However, it also restricted the breadth of our analysis and may have omitted relevant findings presented in those abstracts.

Additionally, most of the included papers were case reports. While these provide valuable insights, they inherently lack the robustness and generalizability of larger, more comprehensive studies. The predominance of case reports limits the ability to draw broader conclusions about the prevalence and severity of gastrointestinal complications post-COVID-19 infection. Furthermore, there is a lack of detailed information regarding the outcomes of the gastrointestinal disorders reported. Many studies did not follow patients longitudinally to assess the resolution or progression of symptoms. This gap in information makes it challenging to understand the long-term impact of these complications.

Finally, the relationship between gastrointestinal complications and the severity of COVID-19, as well as the influence of previous comorbidities and other risk factors, was not consistently addressed across studies. This inconsistency limits our ability to fully elucidate the potential risk factors and underlying mechanisms contributing to these complications. In conclusion, while our systematic review provides important insights into gastrointestinal complications following COVID-19, the aforementioned limitations highlight the need for more comprehensive and longitudinal studies to better understand these associations and outcomes.

## 5. Conclusions

Our systematic review highlights a significant association between COVID-19 and the incidence of gastrointestinal (GI) disorders. The studies reviewed indicate that a wide range of GI complications, including infections, inflammatory conditions, vascular disorders, and structural abnormalities, can arise following COVID-19 infection. Notably, these GI disorders are prevalent among patients who have recovered from COVID-19, suggesting a clear post-infection impact.

The severity of COVID-19 also appears to be directly linked to the severity and variety of reported GI disorders. For example, conditions such as UC and acute pancreatitis have been reported in more severe cases, with inflammation playing a pivotal role. The presence of severe inflammatory responses, such as those seen in mesenteric lymphadenopathy and acute gastritis, further underscores the profound impact of severe COVID-19 on the GI system. Overall, the findings underscore the necessity for healthcare professionals to closely monitor and manage GI complications in patients recovering from COVID-19, particularly those who experienced severe infection. Understanding these associations and the underlying mechanisms is crucial for improving patient outcomes and guiding effective long-term care strategies.

## Data Availability

The data that support the findings of this study are available in the Supplementary Materials of this article.

## Supplementary Materials

The following supporting information can be downloaded at: https://www.mdpi.com/article/10.3390/v16101516/s1, File S1: Database search strategy, Table S1: Demographic and clinical data of the included patients as reported by 24 studies, Table S2: Quality assessment results for case reports and case series using Murad et al. scale [16], Table S3: Quality assessment results for cohort studies using New Castle Ottawa Scale (NOS) [14], Table S4: Quality assessment results for clinical trials using Jadad scale [15], Table S5: post-COVID-19 GI inflammatory conditions, Table S6: post-COVID-19 GI infections, Table S7: post-COVID-19 GI vascular disorders, Table S8: post-COVID-19 GI structural abnormalities, Table S9: other post-COVID-19 GI-diagnosed abnormalities, Table S10: post-COVID-19 GI-undiagnosed conditions. References [18,19,20,21,22,23,24,25,26,27,28,29,30,31,32,33,34,35,36,37,38,39,40,41] are cited in Supplementary Materials.
