# Supplementary material for: Gastrointestinal Sequelae of COVID-19: Investigating Post-Infection Complications—A Systematic Review
Source: Viruses. 2024 Sep 25;16(10):1516. doi: 10.3390/v16101516 (PMC11512271; doi:10.3390/v16101516)
Supplement: Supplementary file 1 [file viruses-16-01516-s001.zip › viruses-3130495-supplementary tables.pdf]

Table S1. Demographic and clinical data of the included patients as reported by 24 studies.

**Author**	**Study Type**<br><br>**County**	**N (total)**<br>**Gender (%M)**	**Type of GI problem**	**Age (years)**<br>**Mean ±SE/**<br>**Median (IQR)**	**Comorbidities**	**COVID-19**<br>**Date/**<br>**duration**<br>**Severity**	**Time of GI problem detection**	**Blood markers**	**Management**	**Follow up time**<br><br>**Outcome**	**QA score**<br><br>**Type of test**
Parrela et al., 2022 [18]	Case report<br><br>Brazil	1 M	1<br><br>Diagnoses<br><br>SMA and jejunal branches Thromboembolism<br><br>Short bowel syndrome<br><br>Signs and symptoms:<br>Nausea<br>Vomiting<br>Fever<br>Bloating<br>Abdominal pain<br>Diarrhea which progressed to obstipation<br>Anasarca<br>Hyporexia<br>Abdominal pain<br>Hypoalbuminemia	62	NR	Date<br>August 2020<br><br>Duration<br>NR<br><br>Severity<br>NR	20 days post COVID-19 hospitalization	NR	Thromboembolictomy<br><br>Enterectomy<br><br>Rivaroxaban 20 mg daily<br><br>Dietary adjustments	2 months after discharge<br><br>Developed:<br>Anasarca<br>Hyperoxia<br>Abdominal pain<br><br>Diagnosed with short bowel syndrome	5<br>Murad et al., scale
Enas et al., 2023 [19]	Case control<br><br>Egypt	210<br><br>(1) COVID-19 history and *Cryptosporidium* + = 49 (23.3% M)	Diagnoses<br><br>*Cryptosporidum* spp. infection	> 18 *(Averages NR)*	(1)<br>*H. pylori* infection (49.9%)<br>Diabetes (20.5%)	NR	NR	NR	NR	NR	7<br>NOS

Author	Study Type<br><br>County	N (total)<br>Gender (%M)	Type of GI problem	Age (years)<br>Mean ±SE/<br>Median (IQR)	Comorbidities	COVID-19<br>Date/<br>duration<br>Severity	Time of GI problem detection	Blood markers	Management	Follow up time<br><br>Outcome	QA score<br><br>Type of test
		(2) COVID-19 history and *Cryptosporidium* - = 161 (76.6% M)<br><br>COVID-19 negative controls n = 220<br><br>Gender NR	Signs and symptoms<br>Chronic diarrhea<br>Vomiting<br>Weight loss<br>Fecal occult blood<br>Elevated inflammatory biomarkers		(2)<br>*H. pylori* infection (16.8%)<br>Diabetes (18.3%)						
Golla, et al., 2023 [20]	Prospective cohort study<br><br>India	920<br><br>Cohort 1 (COVID-19 cases): 320<br>M (50.9%)<br><br>Cohort 2<br>Group A (Age matched controls for cohort 1):320<br>(54.6% M)<br><br>Group B Healthcare workers COVID-19 negative: 280<br>(61.4% M)	50/320 (15.6%)<br><br>Diagnoses<br><br>NR<br><br>Signs and symptoms<br><br>Diarrhea 23 (7.2%)<br><br>Abdominal pain 16 (5.0%)<br><br>Nausea with vomiting 11 (3.4%)<br><br>At 1 month after infection, 36/320 (11.3%) FGID-like symptoms	Cohort 1<br>38.02 ± 11.4	Cohort 1<br>Diabetes 8.4%<br><br>HTN 11.5%<br><br>CAD 2.5%<br><br>CKD 1.2%	Date<br>NR<br><br>Duration<br>NR<br><br>Severity<br>Cohort 1<br>Mild 74.3%<br><br>Moderate 22.1%<br><br>Severe 3.4%	1 month after infection	NR	NR	At 3 months, 27 (8.4%) persisted to have symptoms<br><br>At 6 months, 21 (6.6%) had persistent symptoms<br><br>Of the various reported FGID at 3 months:<br><br>8 (2.5%) IBS, 7 (2.2%) functional diarrhea	8<br>NOS

Author	Study Type<br><br>County	N (total)<br>Gender (%M)	Type of GI problem	Age (years)<br>Mean ±SE/<br>Median (IQR)	Comorbidities	COVID-19<br>Date/<br>duration<br>Severity	Time of GI problem detection	Blood markers	Management	Follow up time<br><br>Outcome	QA score<br><br>Type of test
										6 (1.9%) functional dyspepsia (FD)<br><br>3 (0.9%) functional constipation<br><br>2 (0.6%) FD-IBS overlap<br><br>1 (0.3%) functional abdominal bloating/distention<br><br>Of the 27 patients with persistent symptoms at 3 months:<br><br>8 (29.6%) isolated carbohydrate malabsorption	

Author	Study Type<br>County	N (total)<br>Gender (%M)	Type of GI problem	Age (years)<br>Mean ±SE/<br>Median (IQR)	Comorbidities	COVID-19<br>Date/<br>duration<br>Severity	Time of GI problem detection	Blood markers	Management	Follow up time<br>Outcome	QA score<br>Type of test
										1 (3.7%) 2 positive tests for MAS<br>1 (3.7%) IMO	
Zollner et al., 2022 [21]	(Observational study; Analytical)<br>Austria	46 COVID-19 positive IBD patients<br>(56.5% M)	Diagnoses<br>70% (32/46) of patients with positive qPCR signal in at least 1 segment of the gut.<br>52% (24/46) of patients with immunoreactivity against the viral nucleocapsid phosphoprotein in the small and large intestine<br>NR	44.67 (25.45, 50.58)	Crohn's Disease (22/32 (68.8)<br>UC 8/32 (25.0)<br>IBD unclassified 2/32 (6.2)<br>Heart disease 2/32 (6.2)<br>Diabetes 2/32 (6.2)<br>Lung disease 1/32 (3.1)	Date<br>October 2020 and- February 2021<br>Duration NR<br>Severity<br>Asymptomatic (2.2%)<br>Mild (91.3%)<br>Moderate (4.3%)<br>Severe (2.2%)	Days since PCR)-confirmed SARS-CoV-2 infection and biopsy: 218.50 (94.50, 256.75; Range)	NR	NR	No follow up, single sample	3<br>NOS
Ferreira et al., 2022 [22]	Case-Control Study<br>Brazil	COVID-19 positive cases: 149 (34.23% M)<br>Control group (COVID-19 negative): 71 (28.17% M)	Diagnoses<br>Comparing both groups, significant differences were seen in microbial diversity in post-COVID-19 patients<br>Signs and symptoms	NR	Obesity (38.26%)<br>Systemic arterial hypertension (21%)<br>T2DM (7.4%)	Date<br>October to December 2020 presenting positive COVID-19 test	1-8 months after positive COVID-19 test	Total SARS-CoV-2 Antibodies<br>Asymptomatic: 29.2 ± 49.1	NR	Ranges from 1 month follow up to 8 month follow up	5<br>NOS

Author	Study Type<br><br>County	N (total)<br>Gender (%M)	Type of GI problem	Age (years)<br>Mean ±SE/<br>Median (IQR)	Comorbidities	COVID-19<br>Date/<br>duration<br>Severity	Time of GI problem detection	Blood markers	Management	Follow up time<br><br>Outcome	QA score<br><br>Type of test
			49 had diarrhea		Heart diseases, such as arrhythmias, coronary artery disease and cardiac dispositive (5.4%)<br><br>Chronic lung diseases, including asthma (4.7%)<br><br>Chronic renal disease (2.7%)<br><br>Depression (3.3%)<br><br>Autoimmune diseases (2%)	Duration<br>NR<br><br>Severity:<br>Asymptomatic: 10<br><br>Mild: 117<br><br>Moderate: 10<br><br>Severe: 12		Mild: 68.6 ± 58.8<br><br>Moderate: 101.1 ± 57.5<br><br>Severe: 87.3 ± 43. 4<br><br>CRP (mg/dL)<br><br>Asymptomatic: 0.42 ± 0.27<br><br>Mild: 0.56 ± 0.92<br><br>Moderate: 1.56 ± 1.68<br><br>Severe: 3.22 ± 4.35			
Lee et al., 2021 [23]	Case Report<br><br>South Korea	1 M	Diagnoses<br>Oropharyngeal dysphagia<br><br>Signs and symptoms<br>Dysphagia	73 years	Parkinson's Disease	Date<br>NR<br><br>Duration<br>14 days after testing positive<br><br>Severity<br>NR	21 days after negative PCR	NR	Swallowing therapy<br><br>Enteral feeding	NR	6<br>Murad et al., scale

Author	Study Type<br><br>County	N (total)<br>Gender (%M)	Type of GI problem	Age (years)<br>Mean ±SE/<br>Median (IQR)	Comorbidities	COVID-19<br>Date/<br>duration<br>Severity	Time of GI problem detection	Blood markers	Management	Follow up time<br><br>Outcome	QA score<br><br>Type of test
Banerjee et al., 2023 [24]	Case Report<br><br>India	1 F	<u>Diagnoses</u><br>Intestinal and Mesenteric Mucormycosis<br><br>Pneumoperitoneum with thrombosis of the ileocolic artery<br><br><u>Signs and symptoms</u><br>Abdominal Pain<br>Nausea<br>Vomiting	38 years	Type 2 diabetes Mellitus	<u>Date</u><br>NR<br><br><u>Duration</u><br>2 weeks<br><br><u>Severity</u> NR	After 2 weeks of infection	NR	Exploratory laparotomy<br><br>Ionotropes and vasopressors<br><br>Formation of barrel stoma and a mesh laparostomy<br><br><u>Postoperatively:</u><br>Liposomal amphotericin B (200 mg IV)<br><br>Broad spectrum antibiotics	NR	4<br>Murad et al., scale
Anayat et al., 2022 [25]	Cross Sectional Study<br><br>Pakistan	30<br>(50% M)	<u>Diagnoses</u><br>Dyspepsia (6.7%)<br><br><u>Signs and symptoms</u><br>NR	NR	NR	<u>Date</u><br>NR<br><br><u>Duration</u><br>NR<br><br><u>Severity</u><br>NR	NR	NR	NR	NR	2<br>NOS
AbdurRaheem et al., 2022 [26]	Case report (2 cases)	2 M	<u>Diagnoses</u><br>(1) Abdominal cocoon and SBO	(1) 46	None	<u>Date</u><br>NR	Both cases are recovered patients	NR	NR	NR	5<br>Murad et al., scale

Author	Study Type<br><br>County	N (total)<br>Gender (%M)	Type of GI problem	Age (years)<br>Mean ±SE/<br>Median (IQR)	Comorbidities	COVID-19<br>Date/<br>duration<br>Severity	Time of GI problem detection	Blood markers	Management	Follow up time<br><br>Outcome	QA score<br><br>Type of test
	Country NR		Signs and Symptoms<br>(1) Abdominal pain, nausea, vomiting, constipation			Duration<br>NR<br><br>Severity<br>Severe	(1) 6 months post-COVID-19 recovery				
			Diagnoses<br>Abdominal cocoon<br><br>Signs and symptoms<br>Abdominal pain, nausea, vomiting	(2) 47	None	Date<br>NR<br><br>Duration<br>NR<br><br>Severity<br>Severe	(2) 6 months post COVID-19 diagnosis	NR	NR	NR	
Jain et al., 2022 [27]	Case report<br><br>India	1 F	Diagnoses<br>Mesenteric thrombosis with invasive Mucormycosis<br><br>Signs and symptoms<br>Abdominal pain<br>Constipation	57	Diabetes	Date<br>NR<br><br>Duration<br>NR<br><br>Severity<br>NR	20 days after COVID-19 diagnosis	Hgb 11.8 gm/dl	IV fluids, analgesics, and antibiotics<br><br>Right hemicolectomy<br><br>Inotropic support	Post-operative cardio-pulmonary arrest - Could not be resuscitated	4<br>Murad et al., scale
Cooney et al., 2022 [28]	Retrospective cohort<br><br>UK	122<br>(48% M)	1st survey<br><br>87 (71.3%) total<br><br>Diagnoses<br>Dyspepsia 27 (22.1%)<br><br>Signs and symptoms	NR	IBS (7.4%)	Date<br>Between Feb 4 2020-April 17 2021<br><br>Duration<br>NR<br><br>Severity	NR	NR	NR	NR	2<br>NOS

Author	Study Type<br><br>County	N (total)<br>Gender (%M)	Type of GI problem	Age (years)<br>Mean ±SE/<br>Median (IQR)	Comorbidities	COVID-19<br>Date/<br>duration<br>Severity	Time of GI problem detection	Blood markers	Management	Follow up time<br><br>Outcome	QA score<br><br>Type of test
			Abdominal pain 28 (23%),<br><br>Diarrhea<br>37 (30.3%)<br><br>Constipation 15 (12.3%)<br><br>Nausea 32 (26.2%)			36% required ICU admission					
	Retrospective Cohort<br><br>UK	48<br>(48% M)	<u>6-month follow-up survey</u><br><br><u>Diagnoses</u><br>Dyspepsia 14 (29.2%)<br><br><u>Signs and symptoms</u><br>New abdominal pain 14 (29.2%)<br><br>Diarrhea 9 (18.8%)<br><br>Constipation 5 (10.4%)<br><br>Nausea 5 (10.4%)	NR	NR	<u>Date</u><br>Between Feb 4 2020-Apriil 17 2021<br><br><u>Duration</u><br>NR<br><br><u>Severity</u><br>16.7 % needed ICU	6 months after infection	NR	NR	NR	
Sandal et al, 2023 [29]	Retrospective study (Cohort)<br><br>Turkey	8 Cases<br>(37.5% M)	<u>Signs and symptoms</u><br><br>Vomiting 28.5%<br>Epigastric pain 57.1%<br>Regurgitation 57.1%<br>Anorexia 71.4%	Median 16 (Range: 7-17)	NR	<u>Date</u><br>NR<br><br><u>Duration</u><br>NR<br><br><u>Severity</u>	5.8 months after COVID-19 PCR positivity	NR	Anakinra for the patient who went to ICU for MIS-C<br><br>3 patients received PPI and antacid	NR	9<br>NOS

Author	Study Type<br><br>County	N (total)<br>Gender (%M)	Type of GI problem	Age (years)<br>Mean ±SE/<br>Median (IQR)	Comorbidities	COVID-19<br>Date/<br>duration<br>Severity	Time of GI problem detection	Blood markers	Management	Follow up time<br><br>Outcome	QA score<br><br>Type of test
						NR					
	Retrospective study<br>Cohort<br><br>Turkey	8 controls (with GI symptoms but no previous COVID-19 infection)<br>(37.5% M)	Diagnoses<br>NR<br><br>Signs and symptoms<br><br>Chronic Abdominal Pain	Median - 16 y/o<br>(7-17)	NR	Date<br>NR<br><br>Duration<br>NR<br><br>Severity<br>NR	NR	NR	NR	NR	
Wang et al., 2022 [30]	Case Report	1 F	Diagnoses<br>GERD<br>Gallbladder dyskinesia<br>SIBO<br><br>Signs and symptoms<br>Nausea<br>Abdominal pain<br>Loss of appetite<br>Constipation	55-60	Graves disease<br>HTN<br>HLD<br>prediabetes	Date<br>April 2020<br><br>Duration NR.<br><br>Severity<br>Mild-to-moderate	1 month after COVID-19 diagnosis	Initial (4mo prior):<br>ALP: 173 (IU/L)<br><br>ALT: 15 (U/L)<br>AST: 27 (U/L)<br><br>Serum Leptin: N/A<br><br>Cholesterol: N/A<br><br>HDL: N/A<br><br>LDL: N/A<br><br>Day 9:<br>ALP: 205 (IU/L)	Cholecystectomy<br>Dicyclomine<br>Pantoprazole<br>Ondansetron<br>Paroxetine<br><br>Experimental high fiber diet	Symptoms resolved in 2 months	6<br>Murad et al., scale

Author	Study Type<br><br>County	N (total)<br>Gender (%M)	Type of GI problem	Age (years)<br>Mean ±SE/<br>Median (IQR)	Comorbidities	COVID-19<br>Date/<br>duration<br>Severity	Time of GI problem detection	Blood markers	Management	Follow up time<br><br>Outcome	QA score<br><br>Type of test
								ALT: 14 (U/L)<br>AST: 29(U/L)<br>Serum leptin: 121.2 (ng/mL)<br><br>Cholesterol: 184 (mg/dL)<br>HDL: 76 (mg/dL)<br>LDL: 86 (mg/dL)<br><br>Day 58:<br>ALP: 144 (IU/L)<br>ALT: 12<br>AST: 24<br>serum Leptin: 91.7 (ng/mL)<br><br>Cholesterol: 145 (mg/dL)<br>HDL: 57 (mg/dL)<br>LDL: 70 (mg/dL)			

Author	Study Type<br><br>County	N (total)<br>Gender (%M)	Type of GI problem	Age (years)<br>Mean ±SE/<br>Median (IQR)	Comorbidities	COVID-19<br>Date/<br>duration<br>Severity	Time of GI problem detection	Blood markers	Management	Follow up time<br><br>Outcome	QA score<br><br>Type of test
Abbassi et al., 2021 [31]	Case report<br><br>Texas, US	1 M	<u>Diagnoses</u><br>Bowel perforation<br>Intra-Abdominal adhesions<br><br>Numerous Duodenal and Gastric Ulcers<br><br><u>Signs and symptoms</u><br>Abdominal Pain<br>Nausea Vomiting	34 year old	Morbidly Obese with no comorbidities	<u>Date</u><br>NR<br><br><u>Duration</u><br>26 ICU days<br><br><u>Severity</u><br>Severe	2 months after COVID-19 infection	Reported as unremarkable	Midline Laparotomy requiring adhesiolysis, small bowel resection and anastomosis.	NR	4<br>Murad et al., scale
Natarajan et al., 2022 [32]	Randomized controlled trial	111<br>(59% M)	<u>Diagnoses</u><br>NR<br><br><u>Signs and Symptoms</u><br>Any GI symptoms (54)<br><br>Abdominal pain (13)<br><br>Diarrhea (29)<br><br>Nausea (31)<br><br>Vomiting (5)	Median: 36<br>(IQR = 29–51 years)	NR	<u>Date</u><br>NR<br><br><u>Duration</u><br>Between 25 April and 17 July 2020.<br><br><u>Severity</u><br>mild to moderate COVID-19	Symptoms at enrollment	Overall:<br>Absolute lymphocyte count: 1.5(cells/μL)<br><br>ALT: 30 (IU/L)<br><br>AST: 30 (IU/L)<br><br>Seropositivity at enrollment: n(46)<br><br>WBC: 5.5 (cells/μL)	NR	10-month post COVID-19	2<br>Jadad Scale

Author	Study Type<br><br>County	N (total)<br>Gender (%M)	Type of GI problem	Age (years)<br>Mean ±SE/<br>Median (IQR)	Comorbidities	COVID-19<br>Date/<br>duration<br>Severity	Time of GI problem detection	Blood markers	Management	Follow up time<br><br>Outcome	QA score<br><br>Type of test
			57 (with no GI symptoms)					No GI symptoms at enrollment:<br><br>Overall: Absolute lymphocyte count: 1.6(cells/μL)<br><br>ALT: 28 (IU/L)<br><br>AST: 29 (IU/L)<br><br>Seropositivity at enrollment: n(24)<br><br>WBC: 5.8 (cells/μL)			
Gundogdu et al., 2022 [33]	Case Report	1 M	Diagnoses<br>partial thrombosis in SMV<br><br>Jejunal perforation	58	NR	Date<br>NR<br><br>Duration<br>NR	15 weeks after positive test	WBC: 11.79/uL<br>neutrophils 9.86/uL	-Anticoagulation<br><br>-Small bowel resection	12 days with no further complications<br><br>Outcome:	5<br>Murad et al., scale

Author	Study Type<br><br>County	N (total)<br>Gender (%M)	Type of GI problem	Age (years)<br>Mean ±SE/<br>Median (IQR)	Comorbidities	COVID-19<br>Date/<br>duration<br>Severity	Time of GI problem detection	Blood markers	Management	Follow up time<br><br>Outcome	QA score<br><br>Type of test
			jejunal dilation and thickening<br><br>Abscess formation in abdominal cavity<br><br>Signs and symptoms<br>Abdominal Pain<br>Nausea Vomiting			Severity<br>NR		CRP: 125.4 mg/L<br><br>Lactate: 2.4 mmol/L<br><br>fibrinogen 592.6 mg/dL<br><br>D-dimer 12.77 mg/L<br><br>2 weeks later:<br>CRP: 288.1 mg/L,<br>procalcitonin: 13.25 ng/mL	-Abscess drainage.	survived	
Chancharoenthana et al., 2023 [34]	Observational cohort study<br><br>Thailand	577<br>(48% M)	Diagnoses<br>Anorexia 90.9%<br><br>Signs and symptoms<br>Abdominal pain 62.5%<br>Loss of taste 64%	NR	NR	Date<br>NR<br><br>Duration<br>NR<br><br>Severity<br>NR	NR	NR	None, self resolved in 2-3 months	3 months after initial presentation	6<br>NOS
	Observational cohort study	524 on HD<br>(46.9% M)	Diagnoses<br>Anorexia<br>Dysphagia<br>Peritonitis	48 (+/- 9)	-HTN (92.9%)<br><br>-Diabetes (77.9%)	Date<br>between January 2022 to 31 July 2022	Detected during infection	During COVID-19 infection:	NR	3 months after initial presentation	

Author	Study Type<br><br>County	N (total)<br>Gender (%M)	Type of GI problem	Age (years)<br>Mean ±SE/<br>Median (IQR)	Comorbidities	COVID-19<br>Date/<br>duration<br>Severity	Time of GI problem detection	Blood markers	Management	Follow up time<br><br>Outcome	QA score<br><br>Type of test
	Thailand		<u>Signs and symptoms</u><br>Abdominal pain<br>Diarrhea<br>Constipation Belching<br>Loss of taste		-Heart disease (85.9%)<br>-Pulmonary Disease (9.9%)<br><br>-Hepatic disease (2.7%)	<u>Duration</u><br>NR<br><br><u>Severity</u><br>Mild= 48.1%<br><br>Moderate=26.1 %<br><br>Severe= 25.8%		Baseline Creatinine: 8 +/- 2 mg/dL<br><br>CRP 32+/-14 mg/L<br><br>D-dimer 2749 +/- 578 ng/mL			
	Observational cohort study<br><br>Thailand	34 on PD<br><br>(58.8% M)		71+/-2	HTN (100%)<br><br>Diabetes (88.2%)<br><br>Heart disease (82.4%)<br><br>Pulmonary Disease (14.7%)<br><br>Hepatic disease (11.8%)	<u>Date</u><br>NR<br><br><u>Duration</u><br>Between January 2022 to 31 July 2022<br><br><u>Severity</u><br>Mild= 5.9%<br><br>Moderate=20.6 %<br><br>Severe= 73.5%	Detected during infection	During COVID-19 infection:<br>Baseline Creatinine: 11 +/- 2 mg/dL<br><br>CRP 59+/-11 mg/L<br><br>D-dimer 5339 +/- 786 ng/mL			
	Observational cohort study<br><br>Thailand	19 kidney transplantation<br><br>(57.9% M)		44+/-12	HTN (63.2%)<br><br>Diabetes (31.6%)<br><br>Heart disease (47.4%)	<u>Date</u><br>NR<br><br><u>Duration</u>	Detected during infection	During COVID-19 infection:<br>Baseline Creatinine:	6 of 19 abdominal pain found to be due to mesenteric panniculitis which were all effectively		

Author	Study Type<br><br>County	N (total)<br>Gender (%M)	Type of GI problem	Age (years)<br>Mean ±SE/<br>Median (IQR)	Comorbidities	COVID-19<br>Date/<br>duration<br>Severity	Time of GI problem detection	Blood markers	Management	Follow up time<br><br>Outcome	QA score<br><br>Type of test
					Pulmonary Disease (5.3%)<br><br>Hepatic disease (0%)	between january 2022 to 31 July 2022<br><br><u>Severity</u><br>Mild= 0%<br><br>Moderate=21.1 %<br><br>Severe= 0%		2.5 +/- 0.8 mg/dL<br><br>CRP 17 +/- 9 mg/L<br><br>D-dimer 1699 +/- 175 ng/mL	treated with 8 weeks of oral corticosteroids		
Morita et al., 2023 [35]	Case report<br><br>Japan	1 F	<u>Diagnoses</u><br>UC<br><br>Mesenteric lymphadenopathy<br><br>intestinal inflammation<br><br>erosions & luminal bleeding of colon<br><br><u>Signs and Symptoms</u><br>Abdominal pain<br><br>Diarrhea<br><br>Hematochezia	13	NR	<u>Date</u><br>NR<br><br><u>Duration</u><br>1 month after self limiting COVID-19<br><br><u>Severity</u><br>NR	one month after acquiring COVID-19	Collected period (M - months)<br><br>erythrocyte sedimentation rate (mm/h)<br><br>M2 - 22<br>M2.5 - 21<br>M3.5 - 5<br>M5 - 4<br><br>WBC (#/µL)<br>M1 - 5,400<br>M2 - 12,000<br>M2.5 - 9,700<br>M3.5 - 18,700<br>M5 - 12,000	Antiflatulent (hematochezia subsided; abdominal pain & diarrhea persisted)<br><br>Aminosalicylate therapy (did not work)<br><br>Sulfasalazine (did not work)<br><br>Steroid therapy (achieved remission)	5 months (after the onset of GI symptoms)<br><br>M0 - onset of GI<br>M1<br>M2<br>M2.5<br>M3.5<br>M5	6<br>Murad et al., scale

Author	Study Type<br><br>County	N (total)<br>Gender (%M)	Type of GI problem	Age (years)<br>Mean ±SE/<br>Median (IQR)	Comorbidities	COVID-19<br>Date/<br>duration<br>Severity	Time of GI problem detection	Blood markers	Management	Follow up time<br><br>Outcome	QA score<br><br>Type of test
								CRP (mg/dL)<br>M1 - 0.05<br>M2 - 1.61<br>M2.5 - 0.24<br>M3.5 - <0.03<br>M5 - <0.03<br><br>Lymphocytes (#/μL)<br>M1 - 2,215<br>M2 - 5,880<br>M2.5 - 4,355<br>M3.5 - 5,142<br>M5 - 6,360<br><br>leucine-rich α 2 glycoprotein (μg/mL)<br>M2 - 30.6<br>M5 - 10.2<br><br>IgG<br>(250 mg/dL)<br><br>hemoglobin<br>(11.4 g/dL)<br><br>serum			

Author 	Study Type<br><br>County	N (total)<br>Gender (%M)	Type of GI problem	Age (years)<br>Mean ±SE/<br>Median (IQR)	Comorbidities	COVID-19<br>Date/<br>duration<br>Severity	Time of GI problem detection	Blood markers	Management	Follow up time<br><br>Outcome	QA score<br><br>Type of test
								amyloid A (36.3 µg/mL)<br><br>serum proteinase 3 antineutrophil cytoplasmic antibodies (32.3 U/mL)<br><br>serum iron (8 U/mL)<br><br>ferritin (11.0 µg/mL)<br><br>fecal calprotectin (6,110 mg/kg)<br><br>Elevated levels of IL-6, IL-8, IL-12/IL-23p40, CXCL9, CXCL10 which demonstrate CD8+T cell activation			

Author	Study Type<br><br>County	N (total)<br>Gender (%M)	Type of GI problem	Age (years)<br>Mean ±SE/<br>Median (IQR)	Comorbidities	COVID-19<br>Date/<br>duration<br>Severity	Time of GI problem detection	Blood markers	Management	Follow up time<br><br>Outcome	QA score<br><br>Type of test
								Elevated anti-SARS-CoV-2 spike IgG			
Altuwaijri et al., 2023 [36]	Prospective Study<br><br>Saudi Arabia	50<br>(62% M)	<u>Diagnoses</u><br>19 participants developed IBS (around 25%)<br><br><u>Signs and symptoms</u><br>Nausea<br>Vomiting<br>Diarrhea<br>Abdominal pain	≤50 years: 48<br><br>>50 years: 52	NR	<u>Date</u><br>NR<br><br><u>Duration</u><br>Between june 2020 and june 2021<br><br><u>Severity</u><br>NR	After the recovery (NR specifically; at least 3 months later)	NR	NR	Follow up period of each participant - NR	2<br>NOS
Marinoni et al., 2023 [37]	Phase 3, randomized, double blind, placebo controlled<br><br>Italy	39 (20 in treatment group and 19 in placebo group)<br><br>(52.6% M)	Reported as %: 73.7%<br><br><u>Diagnoses</u><br>Acute pancreatitis<br><br>Hepatic and biliary disease<br><u>Signs and symptoms</u><br>Abdominal pain/discomfort<br><br>Gastroesophageal reflux (acid related)<br><br>Reduced appetite	54.16 (±7.9)	NR	<u>Date</u><br>NR<br><br><u>Duration</u><br>Between Nov. 2022 to May 2023<br><br><u>Severity</u><br>NR	Data collected after:<br>27.0 months (±10.7)<br>Symptom persists for more than 4 weeks	NR	VSL#3 (independent variable)<br><br>relationship between VSL#3 treatment and GI complication:<br><br>Overall - significant (p= 0.0429)<br><br>epigastric pain (p =0.0768)	8 weeks	5<br>Jadad Scale

Author	Study Type<br><br>County	N (total)<br>Gender (%M)	Type of GI problem	Age (years)<br>Mean ±SE/<br>Median (IQR)	Comorbidities	COVID-19<br>Date/<br>duration<br>Severity	Time of GI problem detection	Blood markers	Management	Follow up time<br><br>Outcome	QA score<br><br>Type of test
			Nausea/vomiting<br><br>Diarrhea						IBS (p= 0.9826)<br><br>acid regurgitation - significant (p =0.0159)<br><br>nausea and vomit (p = 0.2343)<br><br>constipation (p =0.0964)		
Tong et al., 2022<br>[38]	Case report	1 M	<u>Diagnoses</u><br>Terminal ileitis<br>Phlegmon adjacent to the mesentery<br><br>Pediatric Inflammatory Multisystem Syndrome<br><br><u>Signs and Symptoms</u><br>Abdominal pain<br>Vomiting	15	None	<u>Date</u><br>NR<br><br><u>Duration</u><br>NR<br><br><u>Severity</u><br>NR	8-weeks Post COVID-19 which was complicated by encephalopath y	CRP: 100 mg/L<br><br>Neutrophili a: 8.51 × 10^9/L<br><br>Ferritin: 118ug/L<br><br>D-dimer: 1.68 mg/L<br><br>Lymphopen ia: 1.17 × 10^9/L	Initial treatment: IV antibiotics,IVIG<br><br>2 weeks: TPN<br><br>Next 2 weeks: gradual diet upgrade which did not resolve symptoms, thus he got ileocecal resection	4-weeks post discharge	6<br>Murad et al., scale

Author	Study Type<br><br>County	N (total)<br>Gender (%M)	Type of GI problem	Age (years)<br>Mean ±SE/<br>Median (IQR)	Comorbidities	COVID-19<br>Date/<br>duration<br>Severity	Time of GI problem detection	Blood markers	Management	Follow up time<br><br>Outcome	QA score<br><br>Type of test
Hosoda et al., 2022 [39]	Case report<br><br>Japan	1 M	Diagnoses<br>Bowel dilatation<br><br>Ascites<br><br>Extensive pneumatosis in gastric intestinalis<br><br>Portal venous gas<br><br>Acute mesenteric ischemia<br><br>Hemorrhagic necrosis in gastric and extensive small bowel mucosa<br><br>Signs and Symptoms<br>Constipation<br>Abdominal distension	82	CAD, DM, HTN	Date<br>NR<br><br>Duration<br>6 weeks post positive result<br><br>Severity<br>Severe	Day 36 after disease onset	Elevated AST and ALT<br><br>D dimer >20 µg/mL<br><br>Lactate 13 mmol/L	Favipiravir and dexamethasone<br><br>Fluid resuscitation and vasopressors	Fatal case: cause of death was extensive gastrointestinal necrosis induced by excessive portal hypertension due to portal and mesenteric vein thrombosis	5<br>Murad et al., scale
Basravi et al., 2023 [40]	Case Report	1 M	Diagnoses:<br>SBO and gangrene due to acute mesenteric ischemia from venous thrombosis<br><br>Peritonitis<br><br>Signs and Symptoms<br>Periumbilical non-radiating pain<br><br>Nausea	68	None	Date<br>NR<br><br>Duration<br>NR<br><br>Severity<br>NR	During COVID-19 infection	CRP: 116 mg/L<br><br>WBC: 11,000<br><br>CPK: 660 µg/L	Side-to-side resection-anastomosis<br><br>Post-surgery: Required ICU care for COVID-19	6 months	5<br>Murad et al., scale

Author	Study Type<br><br>County	N (total)<br>Gender (%M)	Type of GI problem	Age (years)<br>Mean ±SE/<br>Median (IQR)	Comorbidities	COVID-19<br>Date/<br>duration<br>Severity	Time of GI problem detection	Blood markers	Management	Follow up time<br><br>Outcome	QA score<br><br>Type of test
			Vomiting<br><br>No defecation or gas passing for 3 days								
Xu et al., 2023 [41]	Case-Control Study<br><br>USA	COVID-19 Cohort: 154,068 (89.04% M)<br><br>Control 1 (contemporary): 5,638,795 (90.31% M)<br><br>Control 2(historical): 5,859,621 (90.60% M)	Diagnoses<br>GERD<br><br>PUD<br><br>Acute pancreatitis<br><br>Functional dyspepsia<br><br>Acute gastritis<br><br>IBS<br><br>Cholangitis<br><br>Signs and Symptoms<br><br>Signs and Symptoms<br>Constipation<br><br>Abdominal pain<br><br>Diarrhea<br><br>Vomiting<br><br>Bloating	COVID-19 Cohort: 61.42 ± 15.64<br><br>Control 1 (contemporary): 63.46 ± 16.23<br><br>Control 2(historical): 62.89 ± 16.48	(% listed in order of COVID - 19, Contemporary control, and Historical control cohort)<br><br>Cancer: (8.14%, 6.14%, 5.93%)<br><br>CVD: (17.32%, 12.66%, 13.04%)<br><br>Cerebrovascular disease : (1.41%, 0.84%, 0.91%)<br><br>CKD: (19.51%, 16.67%, 15.38%)<br><br>Chronic lung disease: (15.27%, 10.86%, 11.19%)<br><br>Diabetes mellitus type 2: (32.12%, 22.68%, 22.85%)	Date<br>NR<br><br>Duration<br>COVID-19 Cohort: Positive PCR test between March 1st 2020 and January 15th 2021<br><br>Severity<br>Non-hospitalized: 131,915<br><br>Hospitalized: 16,764<br><br>Intensive Care: 5,389	Detected 30 days after positive COVID-19 test until end of follow up (1 year)	(Provided as Absolute burden; difference per 1000 persons at 12 months (95% confidence interval provided)<br><br>COVID-19 cohort compared to Contemporary control (Coagulation):<br>PT>13s : 7.90 (6.99, 8.84)<br>PTT>35s :2.11 (1.63, 2.64)<br>INR>1 : 1.37 (0.93, 1.85)	NR	Median follow up provided with IQR in days:<br><br>COVID-19 Cohort: 408 (interquartile range: 378–500)<br><br>Contemporary Control Cohort: 409 (379–505)<br><br>Historical Control Cohort: 409 (379–504)	8<br>NOS

Author	Study Type<br><br>County	N (total)<br>Gender (%M)	Type of GI problem	Age (years)<br>Mean ±SE/<br>Median (IQR)	Comorbidities	COVID-19<br>Date/<br>duration<br>Severity	Time of GI problem detection	Blood markers	Management	Follow up time<br><br>Outcome	QA score<br><br>Type of test
					HLD: (62.08%, 49.08%, 51.36%)			COVID-19 cohort compared to Contemporary control (Liver and biliary tree function **tests):** Albumin<3.5 g/dL: 20.90 (19.27, 22.57)<br><br>ALT>35 U/L: 16.39 (14.60, 18.22)<br><br>Total protein<6.0 g/dL: 14.54 (13.26, 15.85)<br><br>AST>35 U/L: 10.69 (9.33, 12.10)			

Author	Study Type<br><br>County	N (total)<br>Gender (%M)	Type of GI problem	Age (years)<br>Mean ±SE/<br>Median (IQR)	Comorbidities	COVID-19<br>Date/<br>duration<br>Severity	Time of GI problem detection	Blood markers	Management	Follow up time<br><br>Outcome	QA score<br><br>Type of test
								LDH>100 U/L: 10.22 (9.17, 11.31)<br><br>CRP>0.8 mg/dL: 9.43 (8.49, 10.40)<br><br>ALP>92 U/L: 9.21 (7.94, 10.52)<br><br>Total bilirubin>1.2 mg/dL: 7.40 (6.11, 8.73)<br><br>GGT>30 U/L: 3.25 (2.54, 3.99)<br>Direct bilirubin>0.3 mg/dL: 1.43 (0.98, 1.92)<br><br>Lipase>300 U/L: 0.54 (0.30, 0.82)			

Author	Study Type<br><br>County	N (total)<br>Gender (%M)	Type of GI problem	Age (years)<br>Mean ±SE/<br>Median (IQR)	Comorbidities	COVID-19<br>Date/<br>duration<br>Severity	Time of GI problem detection	Blood markers	Management	Follow up time<br><br>Outcome	QA score<br><br>Type of test
								Amylase>390 U/L: 0.07 (0.01, 0.16)<br><br>COVID-19 cohort compared to Historical control (Coagulation): PT>13s : 4.02 (3.12, 4.97)<br><br>PTT>35s: 1.25 (0.77, 1.78)<br>INR>1: 0.70 (0.26, 1.18)<br><br>COVID-19 cohort compared to historical control (Liver and biliary tree function tests): Albumin<3.			

Author	Study Type<br><br>County	N (total)<br>Gender (%M)	Type of GI problem	Age (years)<br>Mean ±SE/<br>Median (IQR)	Comorbidities	COVID-19<br>Date/<br>duration<br>Severity	Time of GI problem detection	Blood markers	Management	Follow up time<br><br>Outcome	QA score<br><br>Type of test
								5 g/dL: 16.71 (15.08, 18.38)<br><br>ALT>35 U/L: 14.08 (12.29, 15.91)<br>Total protein<6.0 g/dL: 12.42 (11.15, 13.73)<br><br>AST>35 U/L : 9.98 (8.61, 11.38)<br><br>LDH>100 U/L: 11.67 (10.62, 12.76)<br><br>CRP>0.8 mg/dL: 10.67 (9.74, 11.65)			

Author 	Study Type<br><br>County	N (total)<br>Gender (%M)	Type of GI problem	Age (years)<br>Mean ±SE/<br>Median (IQR)	Comorbidities	COVID-19<br>Date/<br>duration<br>Severity	Time of GI problem detection	Blood markers	Management	Follow up time<br><br>Outcome	QA score<br><br>Type of test
								ALP>92 U/L: 7.85 (6.58, 9.16)<br><br>Total bilirubin>1.2 mg/dL: 6.30 (5.01, 7.62)<br><br>GGT>30 U/L: 2.08 (1.37, 2.82)<br><br>Direct bilirubin>0.3 mg/dL: 1.48 (1.03, 1.96)<br><br>Lipase>300 U/L: 0.14 (-0.10, 0.42)<br><br>Amylase>390 U/L: 0.03 (-0.04, 0.12)			

HTN: Hypertension; CKD: Chronic Kidney Disease; UC: Ulcerative Colitis; AST: Aspartate transaminase; ALT: Alanine transaminase; ALP: Alkaline phosphatase; LDH: Lactate Dehydrogenase; HLD: Hyperlipidemia; GTT: γ-glutamyl transferase; SBO: Small Bowel Obstruction; SIBO: Small Intestine Bacterial Overgrowth; PT: Prothrombin Time; PTT: Partial Thromboplastin Time; INR: International Normalized Ratio; CRP: c-

reactive peptide; PD: Peritoneal Dialysis; HD: Hemodialysis; WBC: White Blood Cell Count; CPK: Creatinine Phospho Kinase; GERD: Gastroesophageal Reflux Disease; PUD: Peptic Ulcer Disease; IBS: Irritable bowel Syndrome; CVD: Cardiovascular Disease; NOS: Newcastle - Ottawa Quality Assessment Scale.

Table S2. Quality assessment results for case reports and case series using Murad et al. scale [16].

Study	Score/6
Parrela et al. [18]	5
Lee et al. [23]	6
Banerjee et al. [24]	4
AbdurRaheem et al. [26]	5
Jain et al. [27]	4
Wang et al. [30]	6
Abbassi et al. [31]	4
Gundogdu et al. [33]	5
Morita et al. [35]	6
Tong et al. [38]	6
Hosoda et al. [39]	5
Basravi et al. [40]	5

Table S3. Quality assessment results for cohort studies using New Castle Ottawa Scale (NOS) [14].

Study	Score/8
Enas et al. [19]	7
Golla, et al. [20]	8
Zollner et al. [21]	3
Ferreira et al. [22]	5
Anayat et al. [25]	2
Cooney et al. [28]	2
Sandal et al. [29]	9
Chancharoenthana et al. [34]	6
Altuwaijri et al. [36]	2
Xu et al. [41]	8

Table S4. Quality assessment results for clinical trials using Jadad scale [15].

Study	Score/5
Natarajan et al. [32]	2
Marinoni et al. [37]	5

Table S5. Post-COVID-19 GI inflammatory conditions.

Disorder	Number of studies	Total number of patients	Outcome
UC	1 Study [35]<br>Morita et al., 2023	1/1	Recovered
Mesenteric lymphadenopathy	1 Study [35]<br>Morita et al., 2023	1/1	NR
Acute pancreatitis	2 Studies [37,41]<br>Marinoni et al., 2023**<br>Xu et al., 2023**	NR	NR
Acute gastritis	1 Study [41]<br>Xu et al. 2023**	N/A	NR
MIS-C	1 Study [38]<br>Tong et al., 2022	1/1	Did not recover
Terminal ileitis	1 Study [38]<br>Tong et al., 2022	1/1	Did not recover
Peritonitis	1 Study [40]<br>Basravi et al., 2023	1/1	NR

** No. of patients was not reported in this study. UC: Ulcerative Colitis; MIS-C: MIS-C: Multisystem Inflammatory Syndrome in Children; NR: Not reported.

Table S6. Post-COVID-19 GI infections.

Disorder	Number of studies	Total number of patients	Outcome
Cryptosporidum spp. and *H. Pylori* infection	1 Study [19]<br>Enas et al., 2023	NR	NR

Detected SARS-CoV-2 anti-nucleocapsid in intestines	1 Study [21] Zollner et al., 2022	32/46	NR
Intestinal and mesenteric mucormycosis	1 Study [24] Banerjee et al., 2023	1/1	NR
Positive qPCR signal in at least 1 segment of the gut	1 Study [21] Zollner et al., 2022	32/46	NR
invasive mucormycosis	1 Study [27] Jain et al., 2022	1/1	Did not recover
Phlegmon adjacent to the mesentery	1 Study [38] Tong et al., 2022	1/1	Did not recover
Abscess formation	1 Study [33] Gundogdu et al., 2022	1/1	Recovered

NR: Not Reported; qPCR: quantitative Polymerase Chain Reaction.

Table S7. Post-COVID-19 GI vascular disorders.

Disorder	Number of studies	Total number of patients	Outcome
SMA and jejunal branches thromboembolism	1 Study [18] Parrela et al., 2022	1/1	Did not recover
Pneumoperitoneum with thrombosis of the ileocolic artery	1 Study [24] Banerjee et al., 2023	1/1	NR
Mesenteric thrombosis	1 Study [27] Jain et al., 2022	1/1	Did not recover
Thrombosis in SMV	1 Study [33] Gundogdu et al., 2022	1/1	Recovered
Portal venous gas	1 Study [39] Hosoda et al., 2022	1/1	Did not recover

Acute mesenteric ischemia	2 Studies [39,40]<br>Hosoda et al., 2022<br>Basravi et al., 2023	2/2	1 did not recover<br>1 NR

SMA: Superior Mesenteric Artery; SMV: Superior Mesenteric Vein.

Table S8. Post-COVID-19 GI structural abnormalities.

Disorder	Number of studies	Total number of patients	Outcome
Short bowel syndrome	1 Study [18]<br>Parrela et al., 2022	1/1	Did not recover
SBO	2 Studies [26,40]<br>AbdurRaheem et al., 2022<br>Basravi et al., 2023	2/3	1 recovered<br>1 did not recover
Abdominal cocoon	1 Study [26]<br>AbdurRaheem et al., 2022	2/2	2 recovered
Bowel perforation	2 Studies [31,33]<br>Abbassi et al., 2021<br>Gundogdu et al., 2022	2/2	1 did not recover<br>1 recovered
Intra-abdominal adhesions	1 Study [31]<br>Abbassi et al., 2021	1/1	1 did not recover
Phlegmon adjacent to the mesentery	1 Study [38]<br>Tong et al., 2022	1/1	1 did not recover
Gallbladder dyskinesia	1 Study [30]<br>Wang et al., 2022	1/1	1 recovered
Abscess formation	1 Study [33]<br>Gundogdu et al., 2022	1/1	1 recovered

SBO: Small Bowel Obstruction.

Table S9. Other post-COVID-19 GI diagnosed abnormalities.

Disorder	Number of studies	Total number of patients	Outcome
Oropharyngeal dysphagia	1 Study [23]<br>Lee et al., 2021	1/1	NR
Dyspepsia	3 Studies [25,28,41]<br>Anayat et al., 2022<br>Cooney et al., 2022<br>Xu et al., 2023	67% of 30 pts*<br>27/122	NR
Anorexia	2 Studies [29,34]<br>Sandal et al., 2023<br>Chancharoenthana et al., 2023	530/585	5 NR<br>525 recovered
GERD	3 Studies [30,37,41]<br>Wang et al., 2022<br>Marinoni et al., 2023<br>Xu et al., 2023	1/1	Recovered
SIBO	1 Study [30]<br>Wang et al., 2022	1/1	Recovered
Gastric ulcers	1 Study [31]<br>Abbassi et al., 2021	1/1	Did not recover
Duodenal ulcers	1 Study [31]<br>Abbassi et al., 2021	1/1	Did not recover
Hematochezia	1 Study [35]<br>Morita et al., 2023	1/1	Did not recover
erosions & luminal bleeding of colon	1 Study [35]<br>Morita et al., 2023	1/1	NR
IBS	2 Studies [36,41]<br>Altuwaijri et al., 2023<br>Xu et al., 2023	25%*	NR
Ascites	1 Study [39]<br>Hosoda et al., 2022	1/1	Did not recover
Extensive pneumatosis in gastric intestinalis	1 Study [39]<br>Hosoda et al., 2022	1/1	Did not recover

Hemorrhagic necrosis in gastric and extensive small bowel mucosa	1 Study [39]<br>Hosoda et al., 2022	1/1	Did not recover
PUD	1 Study [41]<br>Xu et al., 2023**	NR	NR

GERD: Gastroesophageal Reflux Disease; SIBO: Small Intestinal Bacterial Overgrowth; IBS: Irritable Bowel Syndrome; PUD: Peptic Ulcer Disease. * only reported as percentage. ** No. of patients was not reported in this study.

Table S10. Post-COVID-19 GI undiagnosed conditions.

Disorder	Number of studies	Total number of patients	Outcome
Nausea	11 Studies [18,20,24,26,28,30–33,36,37]<br><br>Parrela et al., 2022<br>Golla et al., 2023<br>Banerjee et al., 2023<br>AbdurRaheem et al., 2022<br>Cooney et al., 2022<br>Wang et al., 2022<br>Abbassi et al., 2021<br>Natarajan et al., 2022<br>Gundogdu et al., 2022<br>Altuwaijri et al., 2023<br>Marinoni et al., 2023** (No NR)	82/561	2 did not recover<br><br>2 recovered<br><br>Rest NR
Abdominal pain	17 Studies [18,20,24,26–38,41]<br><br>Parrela et al., 2022<br>Golla, et al., 2023<br>Banerjee, et al., 2023<br>AbdurRaheem, et al., 2022	439/1149	5 Did not recover<br><br>16 Recovered<br><br>Rest NR

Disorder	Number of studies	Total number of patients	Outcome
	Jain, et al., 2022<br>Cooney, et al., 2022<br>Sandal, et al., 2023<br>Wang, et al., 2022<br>Abbassi, et al., 2021<br>Natarajan, et al., 2022<br>Gundogdu, et al., 2022<br>Chancharoenthana, et al., 2023<br>Morita, et al., 2023<br>Altuwaijri, et al., 2023<br>Marinoni, et al., 2023**<br>Tong, et al., 2022<br>Xu, et al., 2023**		
Diarrhea	10 Studies [18–20,22,28,32,35–37,41]<br><br>Parrela, et al., 2022<br>Enas, et al., 2023<br>Golla, et al., 2023<br>Ferreira, et al., 2022<br>Cooney, et al., 2022<br>Natarajan, et al., 2022<br>Morita, et al., 2023<br>Altuwaijri, et al., 2023<br>Marinoni, et al., 2023**<br>Xu, et al., 2023**	141/705	2 did not recover<br><br>Rest NR
Hyporexia	3 Studies [18,30,37]<br>Parrela, et al., 2022<br>Wang, et al., 2022<br>Marinoni, et al., 2023**	2/2	1 did not recover<br><br>Rest NR

Disorder	Number of studies	Total number of patients	Outcome
Vomiting	12 Studies [18–20,24,26,29,31–33,36,37,41]<br><br>Parrela, et al., 2022<br>Enas, et al., 2023<br>Golla, et al., 2023<br>Banerjee, et al., 2023<br>AbdurRaheem, et al., 2022<br>Sandal, et al., 2023<br>Abbassi, et al., 2021<br>Natarajan, et al., 2022<br>Gundogdu, et al., 2022<br>Altuwaijri, et al., 2023<br>Marinoni, et al., 2023**<br>Xu, et al., 2023**	25/446	2 did not recover<br><br>3 Recovered<br><br>Rest NR
Weight loss	1 Study [19]<br>Enas, et al., 2023**	NR	NR
FGID-like symptoms	1 Study [20]<br>Golla, et al., 2023	36/320	NR
Dysphagia	1 Study [23]<br>Lee, et al., 2021	1/1	NR
Constipation	6 Studies [26–28,30,39,41]<br>AbdurRaheem, et al., 2022<br>Jain, et al., 2022<br>Cooney, et al., 2022<br>Wang, et al., 2022<br>Hosoda, et al., 2022<br>Xu, et al., 2023**	19/127	1 recovered<br>2 did not recover<br>NR
Regurgitation	1 Study [29]<br>Sandal, et al., 2023	4/8	NR
Loss of taste	1 Study [34]	372/577	372 recovered

Disorder	Number of studies	Total number of patients	Outcome
	Chanchareonthana, et al., 2023		
Abdominal distension/bloating	2 Studies [39,41]<br>Hosoda, et al., 2022<br>Xu, et al., 2023**	1/1	1 did not recover<br>NR

FGID: Functional Gastrointestinal disorders. ** No. of patients was not reported in this study.
